# Protein–peptide docking using CABS-dock and contact information

**DOI:** 10.1093/bib/bby080

**Published:** 2018-09-20

**Authors:** Maciej Blaszczyk, Maciej Pawel Ciemny, Andrzej Kolinski, Mateusz Kurcinski, Sebastian Kmiecik

**Affiliations:** 1 Biological and Chemical Research Centre, University of Warsaw; 2 Faculty of Physics at the University of Warsaw; 3 University of Warsaw

**Keywords:** protein–peptide interaction, molecular docking, flexible docking, protein–peptide complex

## Abstract

CABS-dock is a computational method for protein–peptide molecular docking that does not require predefinition of the binding site. The peptide is treated as fully flexible, while the protein backbone undergoes small fluctuations and, optionally, large-scale rearrangements. Here, we present a specific CABS-dock protocol that enhances the docking procedure using fragmentary information about protein–peptide contacts. The contact information is used to narrow down the search for the binding peptide pose to the proximity of the binding site. We used information on a single-chosen and randomly chosen native protein–peptide contact to validate the protocol on the peptiDB benchmark. The contact information significantly improved CABS-dock performance. The protocol has been made available as a new feature of the CABS-dock web server (at http://biocomp.chem.uw.edu.pl/CABSdock/).

**Short abstract:**

CABS-dock is a tool for flexible docking of peptides to proteins. In this article, we present a protocol for CABS-dock docking driven by information about protein–peptide contact(s). Using information on individual protein–peptide contacts allows to improve the accuracy of CABS-dock docking.

## Introduction

Peptides have an enormous potential as future therapeutics [1]. Rational design of peptide drugs often starts with structure-based investigation of the molecular details of protein–peptide interactions. Since experimental characterization of protein–peptide interactions may be difficult or practically impossible, computational methods, such as molecular docking, can provide valuable support for this stage of drug design [2–4]. Docking of peptides to proteins usually requires specific protocols since the straightforward applicability of standard small-molecule docking programs is generally limited to short peptides [2, 5, 6].

Protein–peptide docking methods face two major issues [2]: sampling efficiency and scoring accuracy. The problem of sampling efficiency arises from the enormous number of possible conformations of a highly flexible peptide. This, combined with the large size of protein–peptide systems and the structural flexibility of protein structures, makes prediction of near-native poses an extreme challenge [7]. Scoring accuracy, on the other hand, is the problem of finding the highest accuracy models within a large pool of predicted conformations. This issue has not been successfully resolved so far, either [8, 9].

Protein–peptide docking approaches can be divided into three categories [2]: (i) template-based docking methods that use known structures of protein–peptide complexes as scaffolds for modeling [10], (ii) local docking methods that use some knowledge about the bound complex (such as protein–peptide contact information [10–12] or approximate localization of the binding site [13–17]) and (iii) global docking methods that do not require any information about the complex structure [18–23] and perform search for both the binding site and the peptide pose. CABS-dock [18, 24] is one of the global docking approaches. While the majority of global docking tools treat the receptor and the peptide as rigid bodies during search for the binding site, CABS-dock allows for their flexibility (unlimited for peptides and significant for protein receptors). In comparison to other docking tools, CABS-dock offers the most effective means for modeling large-scale conformational transitions during docking simulation [25] (see the review on handling protein flexibility in modeling protein interactions [7]).

In general, global docking protocols do not use any knowledge about the binding site, although it is possible to obtain significant enhancement of the quality of global docking by using additional information (even very fragmentary) about the interaction interface [2, 26]. The interaction information may come from experiments [27, 28] or computational methods [12, 23, 29–36]. In this work, we present a CABS-dock extension that enables incorporation of contact information and reporting its performance on the peptiDB benchmark.

## Methods

**Figure 1:**
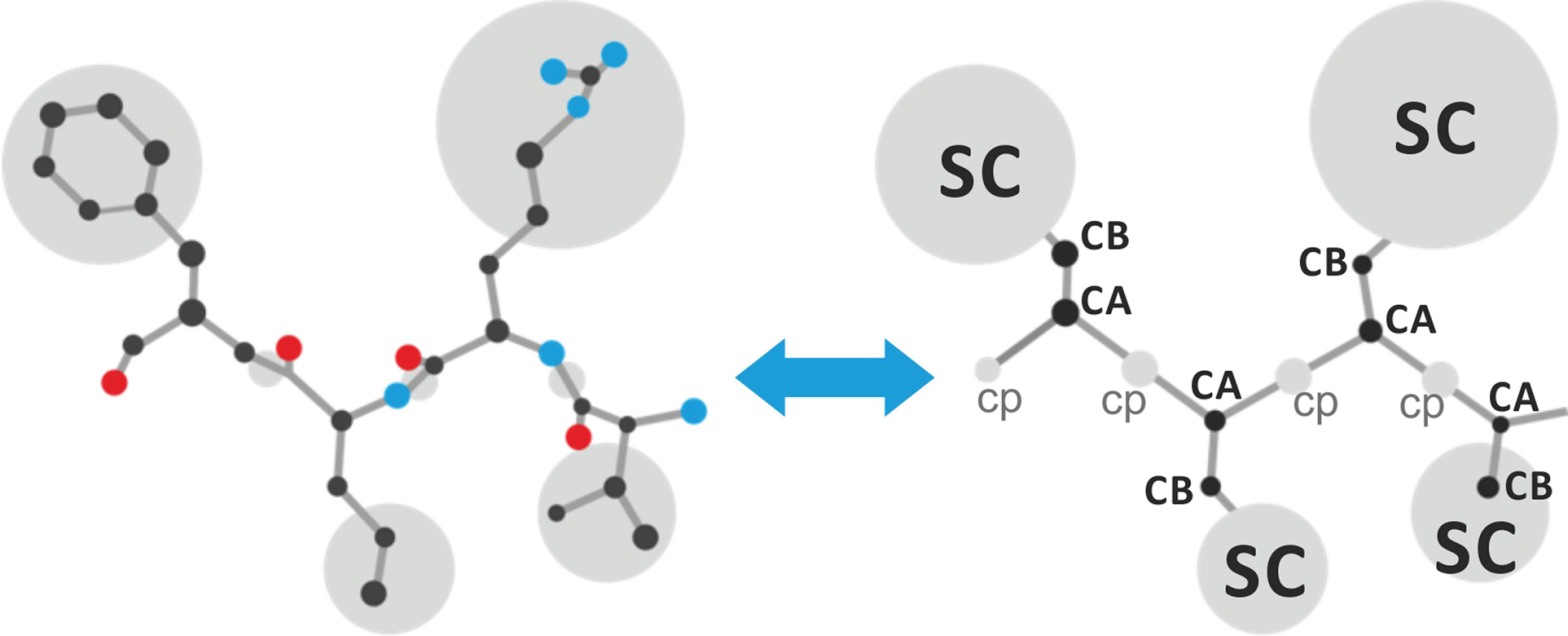
Comparison of all-atom (left) and coarse-grained representation (right) of protein/peptide systems used in CABS-dock docking simulations. CABS-dock assumes the following united atoms or pseudo-atoms representing an amino acid residue: CA, C-beta (CB), SC and center of the peptide bond (cp). In the docking simulation, CABS-dock uses contact information as a distance restraint between the centers of mass of the SC pseudo-atoms with user-defined restraint distance and weight (for details see the Methods section).

CABS-dock uses a CABS coarse-grained model as an efficient simulation engine (the CABS model definition, efficiency and applications to prediction of protein structure, dynamics and interactions have been recently reviewed [37]). In a nutshell, CABS uses coarse-grained representation of peptides and proteins (a single amino acid is represented by up to four atoms or pseudo-atoms, Figure 1), knowledge-based potential (based on statistics derived from known protein structures) and a sampling scheme based on the replica exchange Monte Carlo algorithm.

The CABS-dock protocol for protein–peptide docking [18] consists of four stages:

- docking simulation of a fully flexible peptide and a flexible protein receptor using the CABS model: docking simulation starts from random conformation of a peptide placed in a random position around the protein receptor structure;
- filtering of the models based on CABS protein–peptide interaction energy values (by default, 1000 low-energy models are selected from 10000 conformations generated during docking simulations);
- clustering and scoring of the final models (by default, 10 top-scored models are selected from 1000 low-energy models)
- reconstruction of the final models to all-atom representation (by default, 10 top-scored models are reconstructed). Note that any model selected by a user can be reconstructed to all-atom representation using Modeller [38] (the script is available from the repository of CABS-dock standalone application at https://bitbucket.org/lcbio/cabsdock).

To use the residue–residue contact information, we extended the docking scheme by doing the following:

- introducing a term into the CABS energy function, which works as a distance restraint between selected side chains (SC) (recently, this scheme has been successfully tested on nine modeling cases [39])
- modifying the filtering step preceding the clustering and scoring.

Protein–peptide contact information is introduced into the CABS-energy function as an additional, relatively weak, contact energy term [39], given by the formula

$$ {E}_{\textrm{contact}}(d)=\begin{cases} 0& \textrm{if}\ d\le {D}_0\\ s\left(d-{D}_0\right)& \textrm{if}\ d\gt {D}_0,\end{cases}$$

where $d$ is the distance between the centers of mass of two restrained SC (Figure 1), ${D}_0$ is the distance cutoff and $s$ is the weight of the restraint. If restraint deformation exceeds the user-defined threshold ${D}_0$ (default: 5.0 Å), the energetic penalty linearly increases with the slope defined by the restraint weight, $s$ (default: 1.0). If the measured distance is below the cutoff, peptide motion is not affected. The default parameters in this protocol introduce soft restraints that allow undisturbed flexibility of the peptide within the binding site.

In addition to the new term in the CABS energy function, the filtering step of the CABS-dock docking protocol has been modified. The structures that do not satisfy the user-provided contact criterion are filtered out from the trajectories and excluded before the clustering and scoring step of the protocol.

We tested the contact information-driven CABS-dock protocol on the peptiDB benchmark set [40] of 103 bound and 68 unbound benchmark cases. In each case, the input contact information was a single, randomly chosen native contact derived from the experimental structure stored in Protein Data Bank (PDB). A residue pair was defined to be in contact if the distance between the centers of mass of SC (Figure 1) was less than 5 Å. To analyze the predicted models, we evaluated peptide-RMSD, defined as the Root-Mean Square Deviation (RMSD) of C-alpha (CA) atoms of the peptide, calculated after an optimal superimposition of the native and model receptor structures.

### Submitting contact information using graphical interface of the CABS-dock web server

The contact information-driven docking protocol has been made available as a new feature in the CABS-dock web server (http://biocomp.chem.uw.edu.pl/CABSdock/) . To submit docking tasks with contact information defines a restraint in the ‘contact information’ field using the following format:

<residue1number>:<chainID> <residue2number>:PEP <cutoff value> <restraint weight>,where the cut-off value (${D}_0$, the maximum expected distance between the centers of mass of SC; the default value is 5.0 Å, which has been chosen on the basis of the benchmark tests, and the values of 6.0, 7.0 and 8.0 Å gave qualitatively similar results but the cut-off of 5.0 Å worked best, which is involved with the specifics of the CABS coarse-grained model) and the restraint weight (slope $s$, default value: 1.0) are defined in Formula 1. For example, to introduce a restraint with a cut-off distance of 5.0 Å and restraint weight of 1.0 on residue 1060 of chain C and residue 6 of the peptide, the following string is entered in the appropriate field:

1060:C 6:PEP 5.0 1.0

To introduce multiple restraints, use multiple lines in the ‘contact information’ field. For example, to use the previous restraint together with a second one, imposed on residue 1066 of chain C and residue 7 of the peptide (using the same parameters), the following two lines are typed in the ‘contact information’ field:

1060:C 6:PEP 5.0 1.0

1066:C 7:PEP 5.0 1.0

If the parameters are omitted, the default values will be used. This way all the following three commands will result in the same docking settings:

1060:C 6:PEP 5.0 1.0

1060:C 6:PEP 5.0

1060:C 6:PEP

Note that the contact information used in the docking will be provided under the ‘project information’ tab available from the unique job page [18, 24].

### Submitting contact information using the command line and CABS-dock RESTful service

A docking job with contact information may be also submitted to CABS-dock server via command line using the RESTful service. A detailed tutorial for running CABS-dock from the command line or command line scripts, has been recently provided in the book section [41]. The RESTful service may be used to automate multiple dockings or to incorporate CABS-dock into larger modeling pipeline.

For example, to introduce a restraint with a cut-off distance of 5.0 Å and restraint weight of 1.0 on residue 1060 of chain C (PDB ID of protein receptor: 1AWR:C) and residue 6 of the peptide (peptide sequence: HAGPIA), enter the following string in the command line:

curl -H "Content-Type: application/json" -X POST -d '{"receptor_pdb_code":"1AWR:C", "ligand_seq":"HAGPIA", "contact_information":"1060:C 6:PEP 5.0 1.0"}' http://biocomp.chem.uw.edu.pl/CABSdock/REST/add_job/

To introduce multiple contacts use semicolon as a line separator. For example, to use the previous restraint together with a second one, imposed on residue 1066 of chain C and residue 7 of the peptide (using the same parameters), type the following command in the command line:

curl -H "Content-Type: application/json" -X POST -d '{"receptor_pdb_code":"1AWR:C", "ligand_seq":"HAGPIA", "contact_information":"1060:C 6:PEP 5.0 1.0;1066:C 7:PEP 5.0 1.0"}' http://biocomp.chem.uw.edu.pl/CABSdock/REST/add_job/

### Submitting contact information using CABS-dock standalone application

CABS-dock is also available as a standalone application. CABS-dock standalone combines several tools (for coarse-grained docking, scoring, structural clustering, reconstruction to all-atom representation and docking analysis) into a software package that can be freely customized. CABS-dock standalone uses a similar definition of distance restraints as a web server version (the application source code and tutorials can be accessed from the repository at https://bitbucket.org/lcbio/cabsdock).

## Results

**Figure 2:**
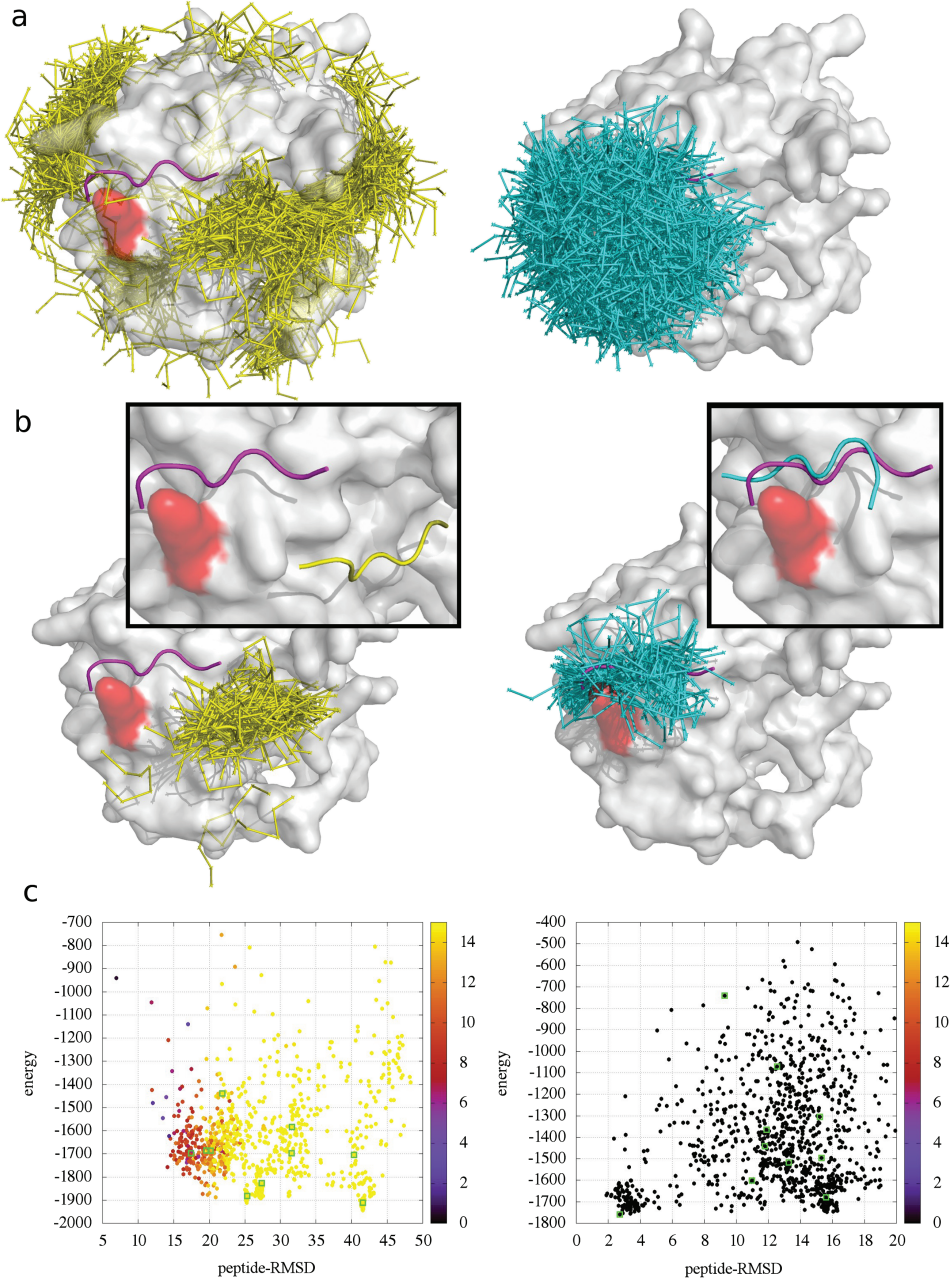
Comparison of CABS-dock docking without contact information (left panel) and with contact information (right panel) for the 1LVM case. The protein residue involved in the contact information, which has been used in docking, is marked in red. (**a**) 1000 top-scored models, (**b**) the best cluster together with the top-scored model in the close-up frame and (**c**) CABS-dock energy versus peptide-RMSD plot, where color of the dots denotes distance (in Angstroms) of the residues used in the contact information above the default cut-off of 5 Å (black color indicates that the distance between the residues is within the cutoff). Visualizations (**a**) and (**b**) show experimental conformation of the peptide (magenta), models from docking without contact information (yellow), models from information-driven docking (cyan) and the receptor surface (white).

As expected, using contact information in CABS-dock global docking leads to a significant improvement of docking accuracy in comparison to CABS-dock docking with default settings [26]. The overview of the differences between the results from the two approaches is presented in Figure 2 using the example of 1LVM (one of the peptiDB cases). In this case, our default global docking procedure was not successful: the peptide-RMSD of the highest accuracy model among 10 000 models, and among the 10 top-scored models, was 5.42 and 13.47 Å, respectively. Adding the contact information improved these values to 1.47 and 2.41 Å. As demonstrated in Figure 2, combination of the new contact potential and the filtering scheme leads to more accurate sampling of the proximity of the binding site (Figure 2A, right) and overall improvement of the quality of produced models (Figure 2C, right).

**Figure 3:**
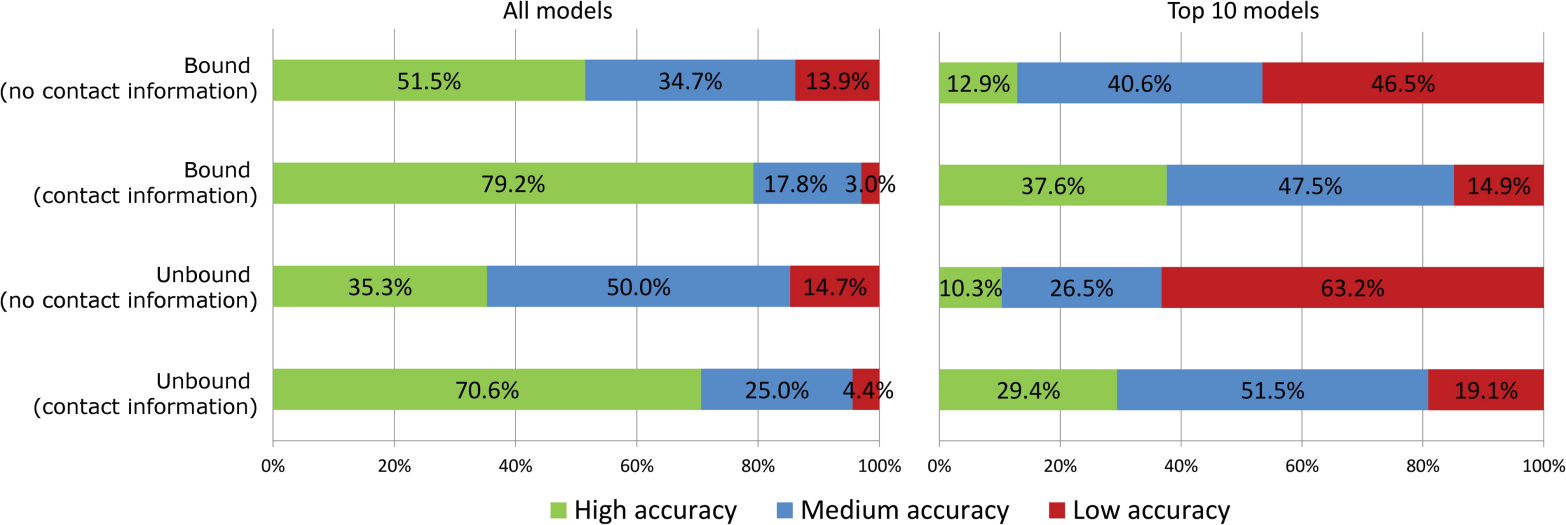
Comparison of CABS-dock performance with contact information and without contact information for 103 bound and 68 unbound benchmark cases. The percentages of high-, medium- or low-accuracy models (quality assessment criteria are given in the text) are reported for the best quality models found in the sets of 10 000 models (all models, left panel) and in the sets of 10 final models (top 10 models, right panel). Detailed results for each modeled complex and each prediction run are available in Supplementary Tables S1 (bound docking cases) and S2 (unbound docking cases). Modeling results for docking without contact information have been taken from our previous work [18].

**Figure 4:**
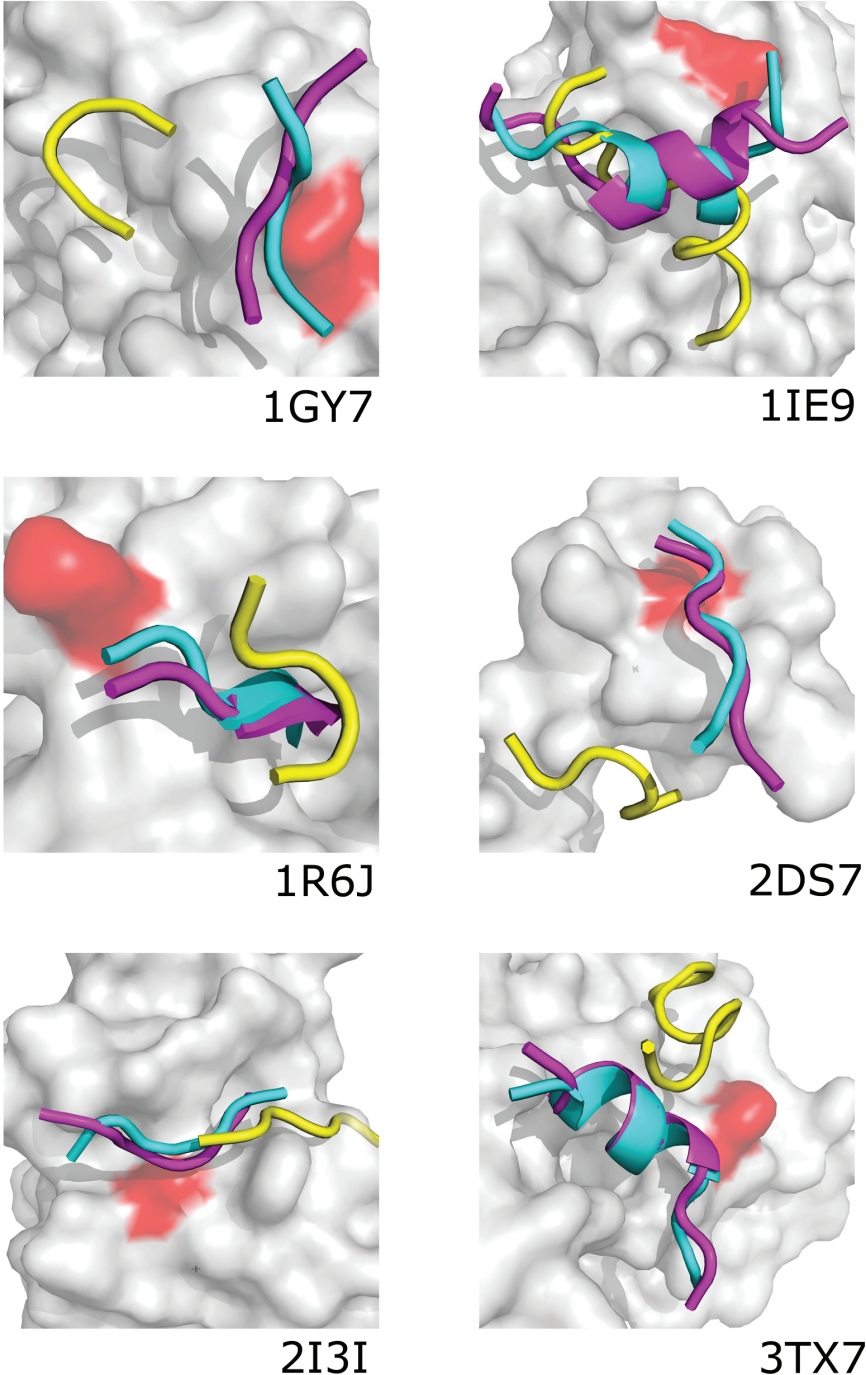
Comparison of top-scored peptide models obtained with contact information (cyan), without contact information (yellow) and experimental structures (magenta). The figure presents the lowest resolution models out of the 10 top-scored models for the example modeling cases (PDB IDs are given in the picture). The following improvement has been noted in terms of peptide-RMSD values (between docking without and with contact information): 1GY7, from 13.71 to 3.21 Å; 1 IE9, from 12.85 to 1.23 Å; 1R6J, from 8.31 to 1.37 Å; 2DS7, from 11.24 to 2.33 Å; 2I3I, from 9.40 to 2.27 Å; 3TX7, from 8.25 to 3.58 Å.

Figure 3 presents the summary of the quality of results obtained for the entire benchmark set. Quality assessment criteria were defined as in the original CABS-dock work [18]: high-accuracy (peptide-RMSD <3 Å), medium-accuracy (3 Å ≤ peptide-RMSD ≤5.5 Å) and low-accuracy (peptide-RMSD >5.5 Å). Overall, in comparison to docking without contact information, the new protocol resulted in an over 3-fold improvement of the fraction of benchmark cases for which the high-quality models were ranked among the 10 top-scored models (Figure 3, right panel). Selected examples of top-scored models obtained with contact information and without contact information are shown in Figure 4. Moreover, the fraction of benchmark cases for which high-accuracy models were generated in the set of all models increased from 51% to 79% of bound cases and from 35% to 70% of unbound cases (Figure 3, left panel).

In some of the benchmark cases, however, there was no significant improvement in comparison to docking without contact information. This lack of improvement can be mainly attributed to hardly (or not at all) accessible binding sites in the input protein structure: the binding site was either localized in a deep pocket (sometimes even inside the protein structure) or covered by a flexible part of the protein (see examples provided in Figures S1 and S2 in the Supplementary Information). Moreover, the results analysis showed that the docking performance may strongly depend on the localization of the residue–residue contact (obviously, a contact that involves a residue localized in the center of the peptide usually works better than the one with a residue close to the peptide ends; Tables S2 and S3 in the Supplementary Information). We observe also a slight dependence of the docking quality on the peptide length (Figure S3 in the Supplementary Information).

The detailed information on the input data used to run the benchmark tests is provided in Table S1, including the information on residue–residue contacts secondary structure information (predicted by PSIPRED method [42]) used in the docking. The peptide-RMSD values obtained in all the runs for the entire benchmark set are listed in Table S2 (bound cases) and Table S3 (unbound cases).

Within this work, we focus on using information on a single protein–peptide contact. However, the presented CABS-dock protocol enables using contact information in different scenarios, depending on the knowledge about the modeled protein–peptide complex. The results of additional docking tests are presented in the Supplementary Information and include docking using more than one residue–residue contact information (Figure S4), ambiguous contact information (with restraints between a single receptor residue and all the peptide residues using a uniform large cut-off distance; Figures S5 and S6), PepSite [30] contact predictions (correct and erroneous) (Figure S7). These additional tests show that using additional or ambiguous or erroneous (but close to correct) contact information can also enhance the CABS-dock prediction accuracy.

**Figure 5:**
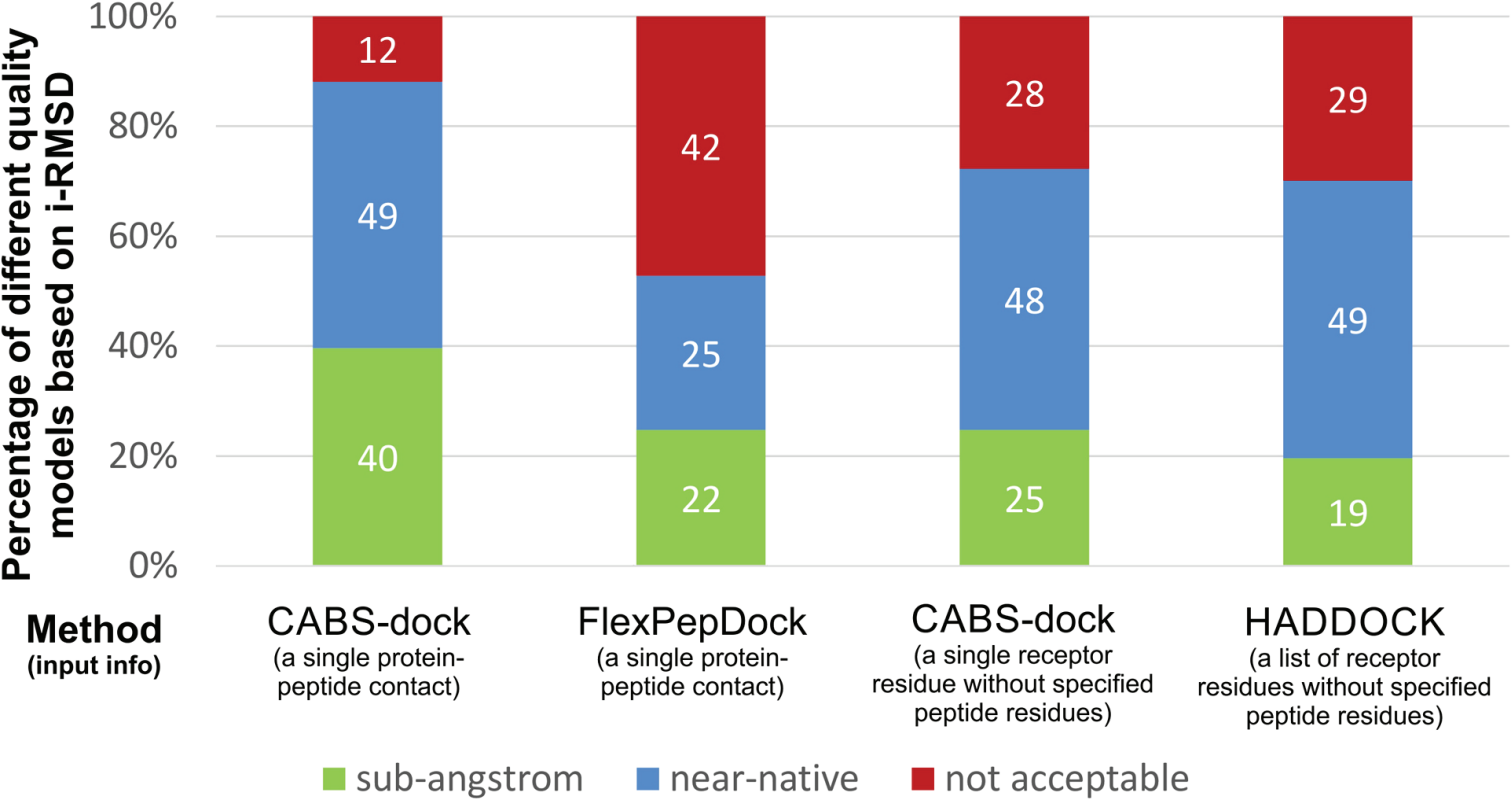
Comparison of CABS-dock (using two kinds of input data), HADDOCK and Rosetta FlexPepDock performance. The figure shows percentage of benchmark cases that fall into different quality categories. HADDOCK and FlexPepDock performance data were taken from the work by Trellet *et al.* [13], CABS-dock results are presented for the highest accuracy model out of the set of 400 top-scored models (400 models were randomly selected from 1000 top-scored models). The quality categories are based on interface RMSD (i-RMSD) values: sub-Angstrom prediction, i-RMSD ≤1 Å; near-native prediction, 1 Å ≤ i-RMSD ≤2 Å; not acceptable, i-RMSD >2 Å. As highlighted in the figure, the presented methods use different kinds of input data; therefore, the comparison is not straightforward. The following protocols are presented: CABS-dock using the information of a single protein–peptide contact, Rosetta FlexPepDock using the information on single protein–peptide contact (the contact information was used in the preparation of input complex structures), CABS-dock using a single receptor residue without specified peptide residues (using ambiguous contact information that is restraints to all peptide residues with the cut-off distance defined as the number of peptide residues plus 12, in Angstroms, this uniform cutoff has been chosen based on the docking test runs presented in Figures S5 and S6) and HADDOCK using a list of receptor residues without specified peptide residues.

Furthermore, we compared docking results with those from HADDOCK and Rosetta FlexPepDock local docking tools (based on data and accuracy criteria from the work of Trellet *et al.* [13]). The comparison is shown in Figure 5 and indicates that CABS-dock performs similarly or better than the other tools. Note, however, that the comparison is not straightforward since HADDOCK and FlexPepDock use different approaches to guide the docking and different input data. Namely, HADDOCK uses restraints based only on a list of receptor residues without specified peptide residues, while FlexPepDock simulations started from an extended peptide structure anchored at a known anchor position (protein–peptide contact).

## Conclusions

In this work, we demonstrated that the incorporation of the contact information into the CABS-dock protein–peptide docking leads to a significant increase of prediction quality. The contact information can be deduced from experimental data [28] (for example from NMR or mutagenesis experiments), structures of similar protein–peptide complexes (template-based modeling) or computational predictions of protein–peptide contacts that may include predictions of the binding site [29–32, 36], key interactions [33], peptide hot-spot analysis [34] and coevolution and conservation analyses [12, 35].

It is important to note that the CABS-dock input of contact information can take into account various levels of accuracy (controlled by the restraint parameters in Formula 1). The restraint can pull the peptide to the vicinity of the binding site, where the generic CABS force field can take over. Therefore, even approximate data can be used in CABS-dock modeling procedures that include predictions of protein–peptide complexes [18, 24], protein–protein complexes [43] or dynamics simulations of intermediate complexes formed during the binding of the peptide [25, 44].

The presented protocol for CABS-dock docking with contact information can be accessed via a graphical user interface within the CABS-dock web server, command line execution using the CABS-dock RESTful web service or CABS-dock standalone application. The RESTful service and CABS-dock standalone application enables easy incorporation of the CABS-dock protocol within high-throughput modeling pipelines that integrate different tools.

**Key Points**

- CABS-dock is a tool for flexible docking of peptides to proteins.
- In this article, we present a protocol for CABS-dock docking driven by information about protein–peptide contact(s). Using information on individual protein–peptide contacts allows improving the accuracy of CABS-dock docking.
- The protocol for protein–peptide docking using CABS-dock and contact information is available within the CABS-dock web server.

## Supplementary Material

Brief_in_Bioinfo_Suppl_FINAL_11_bby080
