# Supplementary material for: Protein–peptide docking using CABS-dock and contact information
Source: Brief Bioinform. 2018 Sep 20;20(6):2299–305. doi: 10.1093/bib/bby080 (PMC6954405; doi:10.1093/bib/bby080)
Supplement: Brief_in_Bioinfo_Suppl_FINAL_11_bby080 [file brief_in_bioinfo_suppl_final_11_bby080.docx]

**Supplementary Information – Using contact info in CABS-dock protein-peptide docking**

**Figure S1. 2IOG: unsuccessful docking case that requires modeling of significant receptor flexibility.** In this case, the binding site is occupied by the N-terminal region of the protein in the unbound structure. The figure presents the receptor surface (white) together with conformation of the N-terminal region in unbound conformation (gray), bound conformation (blue) and bound peptide conformation (magenta).

**
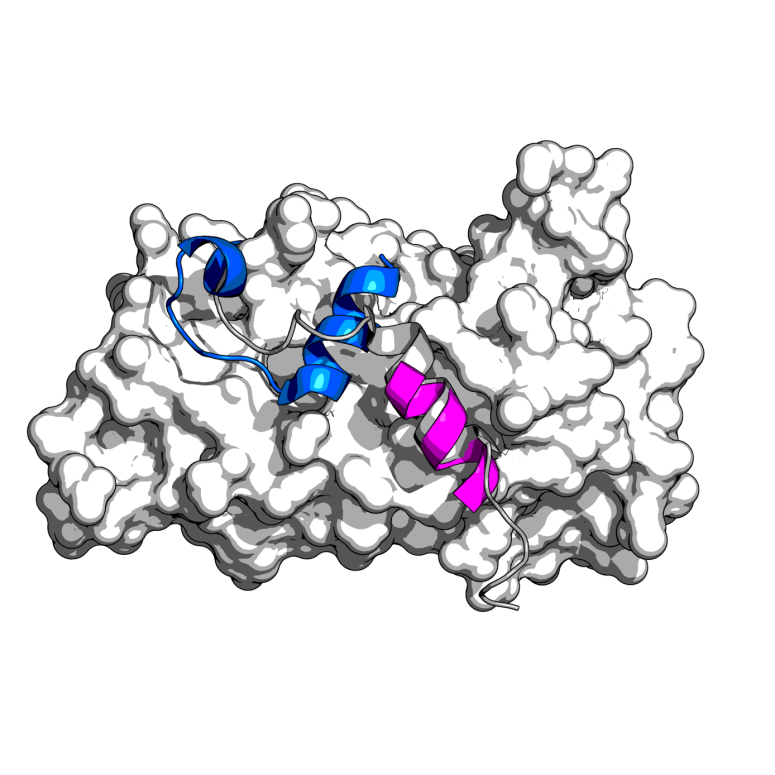
**

**Figure S2. 1U00: unsuccessful docking case with the binding site localized within a deep pocket.** Even though the restraint is satisfied, the peptide did not enter the deep binding pocket. The figure presents the receptor surface (white), 10 top-ranked models (cyan), the native conformation (magenta) and the receptor residue involved in the residue-residue contact used in the docking (red).


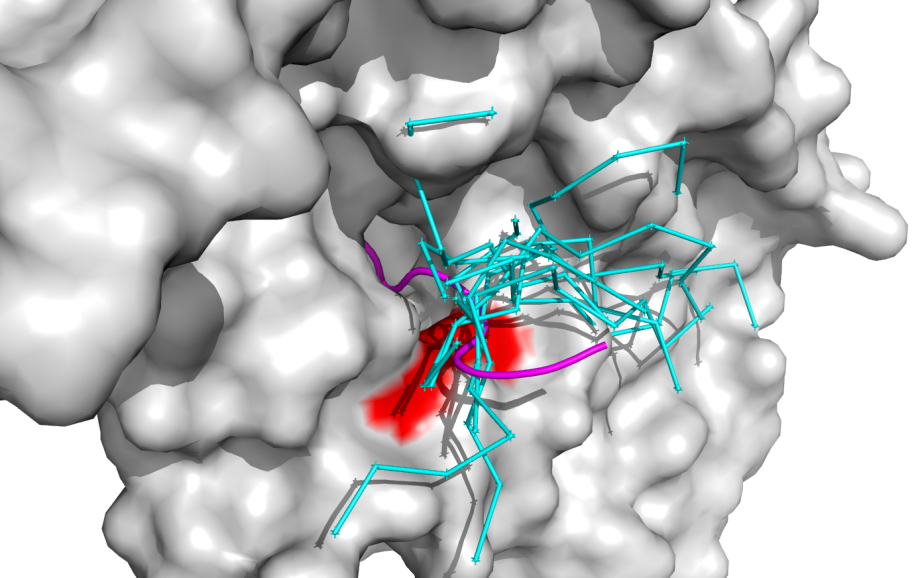


**Figure S3.** **Peptide length vs. RMSD of the highest accuracy model (out of 10’000 of models).**


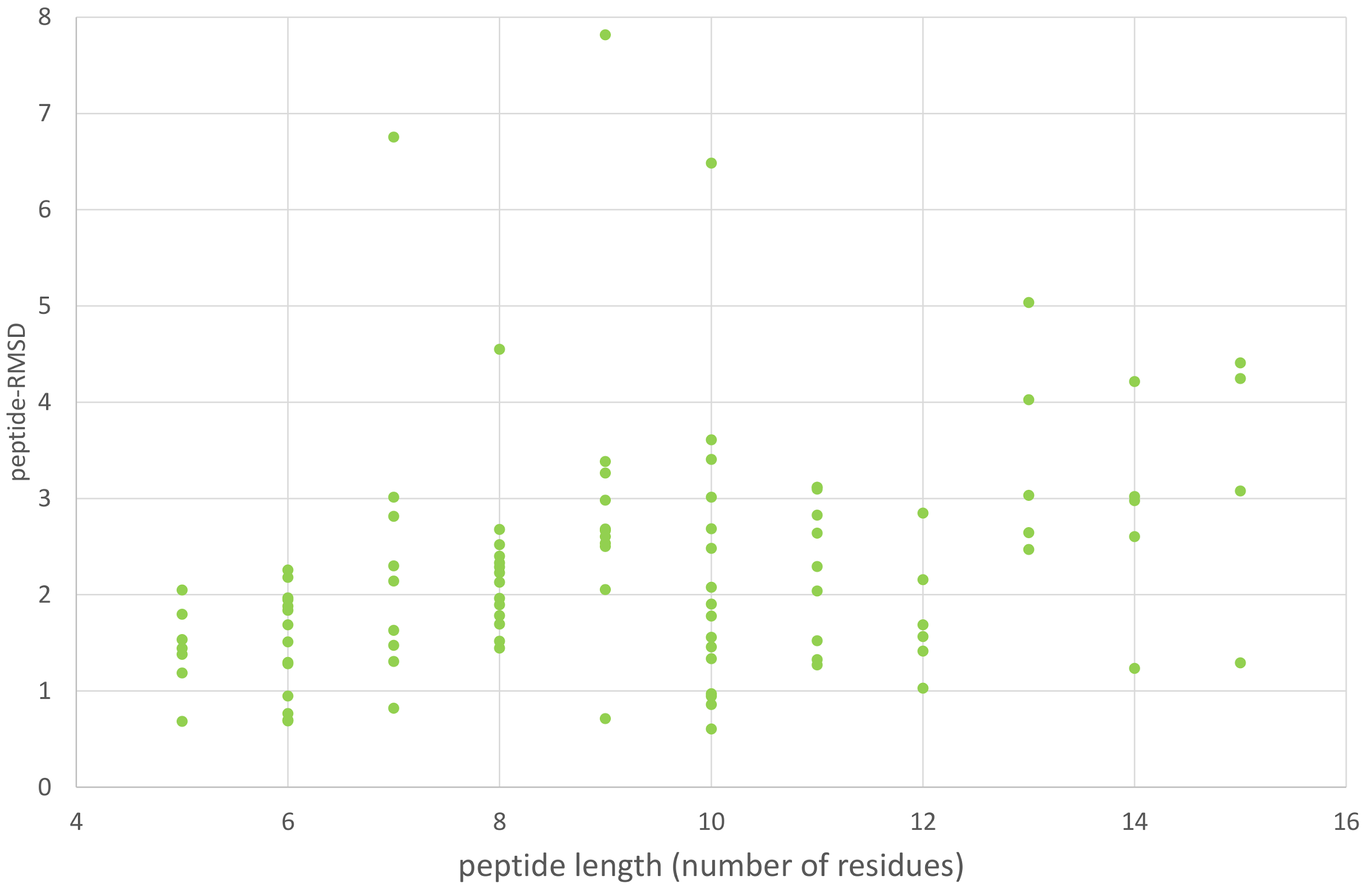


**Figure S4. Comparison of the CABS-dock performance depending on number of provided residue-residue contacts.** The figure compares docking using: no contact information and information about one, two or three randomly selected contacts (number of contacts is given in brackets). As presented in the figure, additional contact information significantly enhances the prediction accuracy (for the best models among 10 top scored). The accuracy criteria are the same as provided in Figure 3 of the manuscript.

*
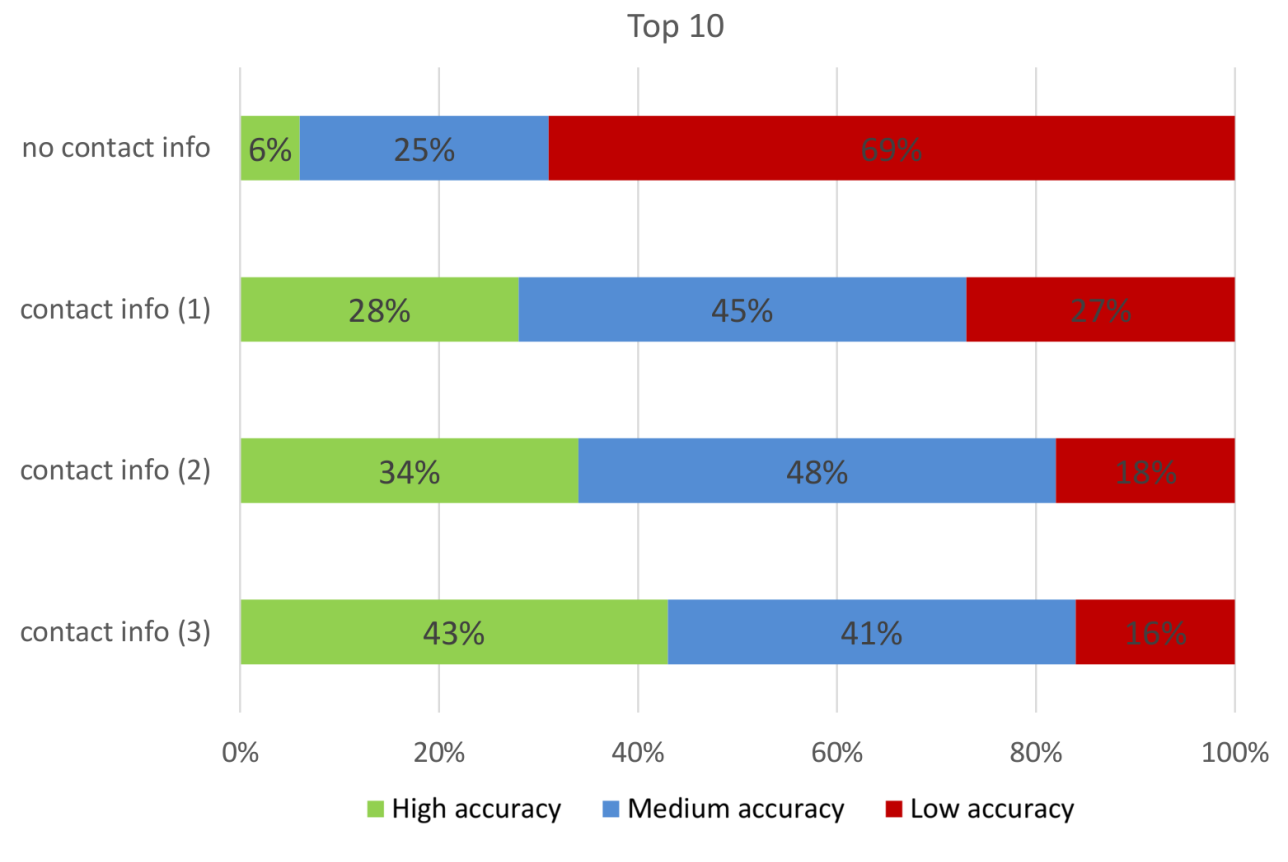
*

**Figure S5. Docking with ambiguous contact information.** The figure presents three scenarios of using CABS-dock protocol: (1) default setting without any contact information, presented for reference, (2) performance using contact information about a single pair of receptor and peptide residues, presented for reference, and (3) performance using ambiguous contact information. To mimic a situation in which only ambiguous contact information is available, or only a single interacting receptor residue is known, we set restraints between a single randomly selected receptor residue and all the peptide residues with a uniform large cut-off distance. The cut-off was set to the maximum distance between any peptide residue and the selected receptor residue measured in the native complex structure (to estimate cut-off distance see **Figure S6**). As presented in the figure, ambiguous contact information significantly enhances the prediction accuracy in comparison to docking with no contact information. The accuracy criteria are the same as provided in the Figure 3 of the manuscript.


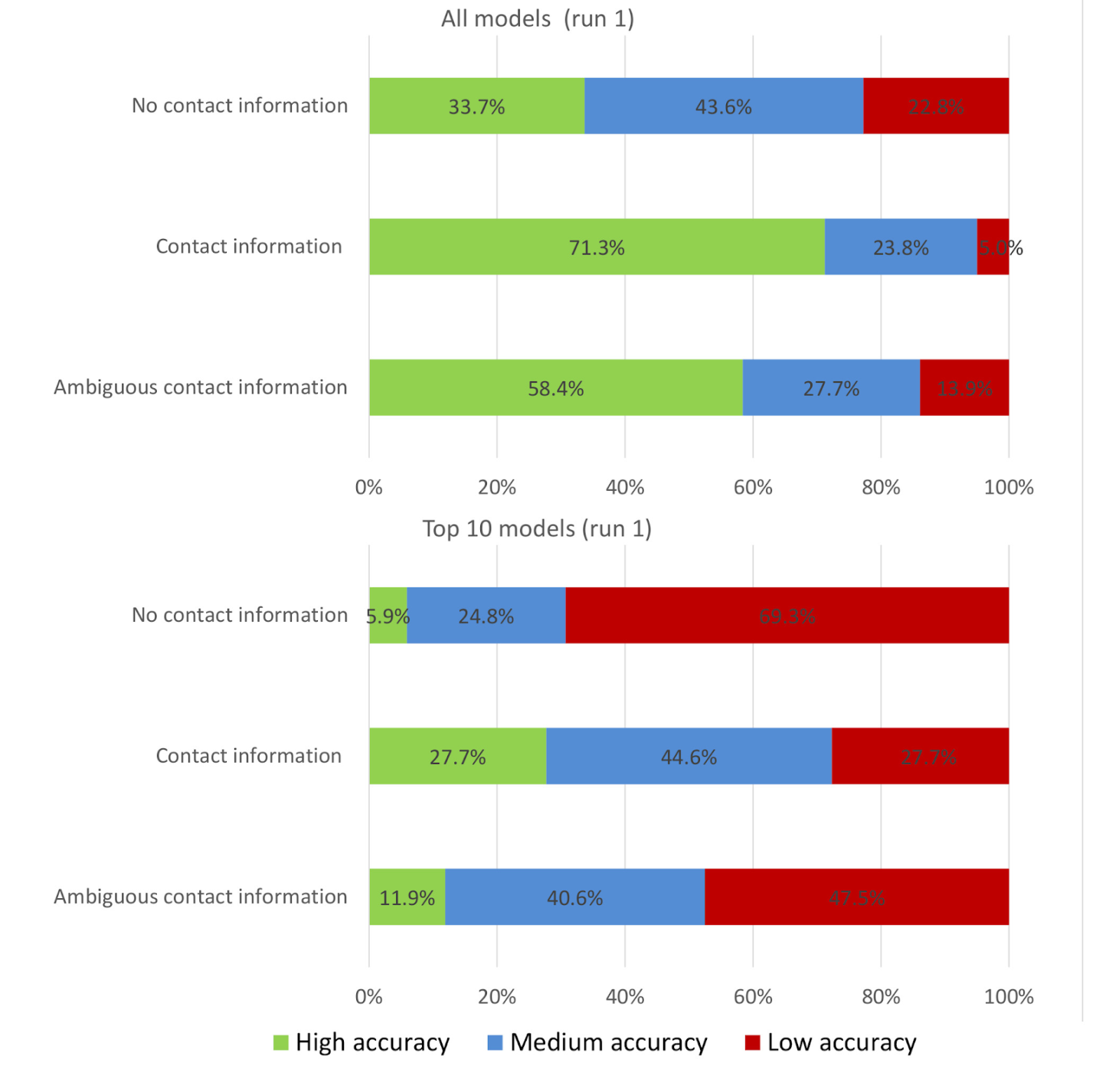


**Figure S6. Peptide length versus restraint cut-off distance used in docking with ambiguous contact information.** The restraint length is the maximum distance between any peptide residue and the selected receptor residue in the native complex structure. This plot may be used to estimate the cut-off distance for the restraints set on the all peptide residues, in the case when only the interacting residue of protein receptor is known. The details of procedure are described in **Figure S5**.


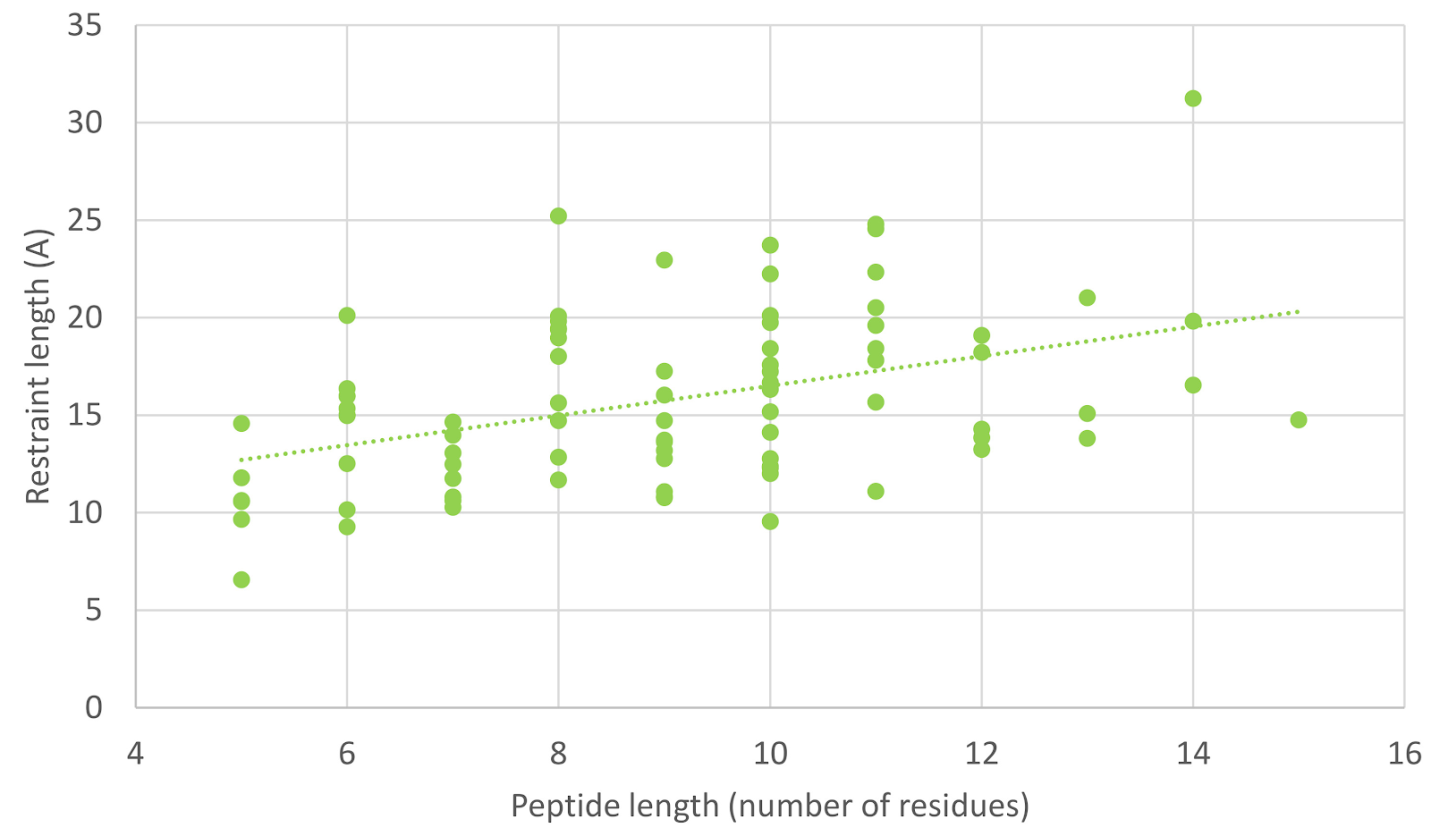


**Figure S7. CABS-dock results using PepSite method as a source of contact information**. Contacts were derived based on the predictions of the binding site by the PepSite method (Trabuco LG, Lise S, Petsalaki E et al. PepSite: prediction of peptide-binding sites from protein surfaces, Nucleic Acids Res 2012;40:W423-427). PepSite, for given receptor structure and peptide sequence, provides set of 10 predictions of the peptide residues which are expected to be in contact. Additionally, method provides PDB file containing predicted positions of contacting peptide residues. Based on this information, we derived list of contacts which were used as the input for the CABS-dock method. Predicted contacts were verified using experimental structure of the complex and were divided into two groups (correct and incorrect contacts). The figure presents the data for 21 cases from the bound benchmark set which satisfies following criteria: (1) at least one prediction (out of ten for each target) were scored by the PepSite as significant (p-value<0.25) and (2) set of ten predictions consist of at least one correctly predicted contact. As presented in the Figure, the more correct the input data (taken from the PepSite predictions), the better performance can be expected. Moreover, in many cases, even substantial fraction of incorrect contacts didn’t prevent the CABS-dock from achieving high accuracy (L-RMSD < 3 A) or medium accuracy models (3 A < L-RMSD < 5.5 A). Inspection of PepSite predictions used in CABS-dock docking showed also that in many cases PepSite provided incorrect but close to correct contact data (i.e. contacts in the closest neighborhood to native contacts).


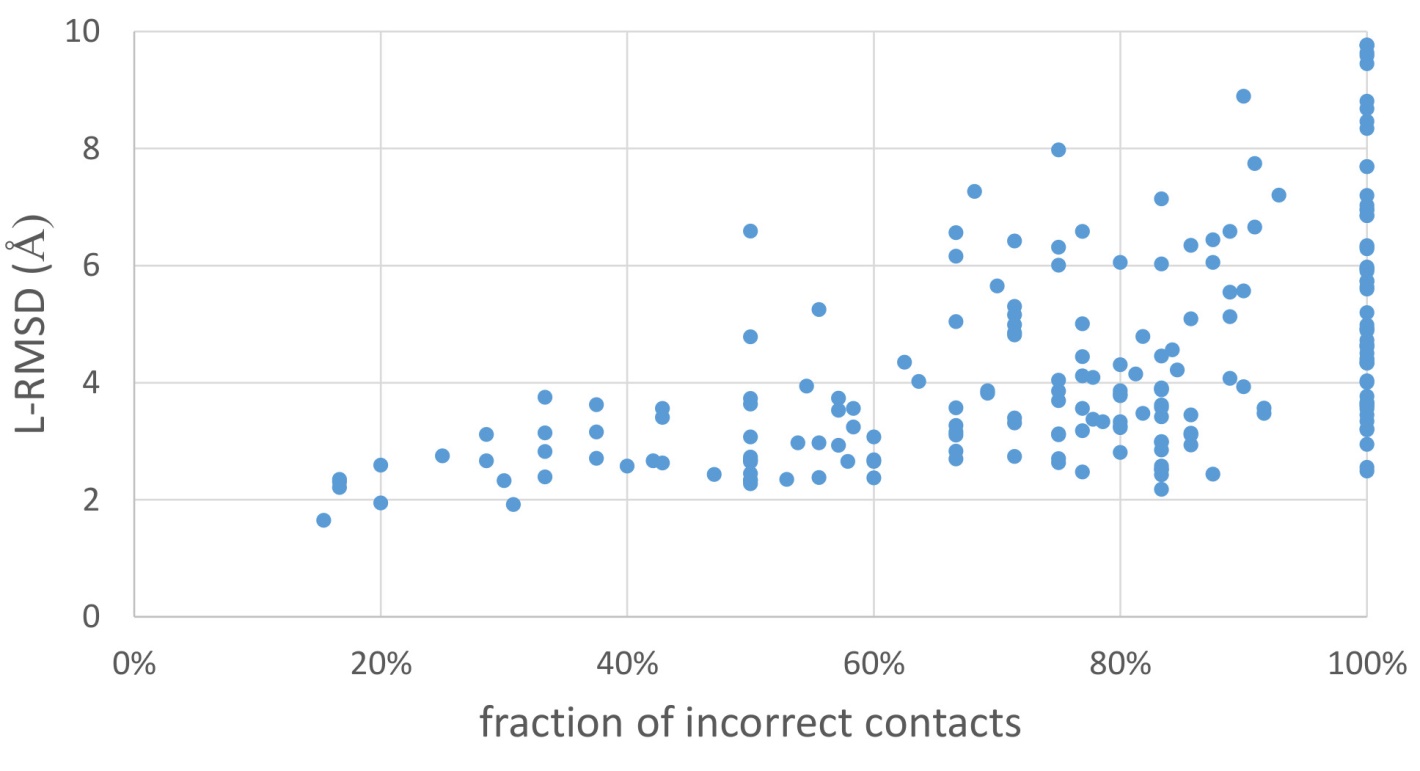


**Table S1. Benchmark set.** Input data for all the benchmark cases in 3 independent prediction runs (shown in separate columns).

| BOUND FORM | | | | | | UNBOUND FORM | | | | | | PEPTIDE | | |
| --- | --- | --- | --- | --- | --- | --- | --- | --- | --- | --- | --- | --- | --- | --- |
|  | RECEPTOR | | CONTACT INFO | | |  | RECEPTOR | | CONTACT INFO | | | SEQUENCE | SECONDARY STRUCTURE | NR AA |
| PDB | CHAIN | NR AA | RUN 1 | RUN 2 | RUN 3 | PDB | CHAIN | NR AA | RUN 1 | RUN 2 | RUN 3 |  |  |  |
| 1AWR | C | 164 | 1060:C 6:PEP | 1101:C 2:PEP | 1063:C 3:PEP | 2ALF | A | 164 | 126:A 4:PEP | 102:A 2:PEP | 102:A 2:PEP | HAGPIA | CCCCCC | 6 |
| 1CE1 | HL | 431 | 104:H 3:PEP | 91:L 6:PEP | 94:L 6:PEP | 1UM5 | HL | 433 | 100:H 2:PEP | 103:H 2:PEP | 103:H 2:PEP | GTSSPSAD | CCCCCCCC | 8 |
| 1CKA | A | 57 | 166:A 8:PEP | 166:A 9:PEP | 183:A 6:PEP | 2DVJ | A | 228 | 25:A 9:PEP | 168:A 7:PEP | 141:A 3:PEP | PPPALPPKK | CCCCCCCCC | 9 |
| 1CZY | C | 168 | 455:C 3:PEP | 467:C 3:PEP | 399:C 4:PEP | 1CZZ | C | 187 | 399:C 4:PEP | 465:C 7:PEP | 410:C 4:PEP | PQQATDD | CEECCCC | 7 |
| 1D4T | A | 104 | 68:A 9:PEP | 52:A 5:PEP | 17:A 3:PEP | 1D1Z | C | 101 | 3094:C 9:PEP | 3093:C 11:PEP | 3067:C 8:PEP | KSLTIYAQVQK | CCCEEEECECC | 11 |
| 1DDV | A | 104 | 71:A 2:PEP | 74:A 6:PEP | 71:A 6:PEP | 1I2H | A | 138 | 1071:A 2:PEP | 1072:A 6:PEP | 1074:A 6:PEP | TPPSPF | CCCCCC | 6 |
| 1DKX | A | 219 | 436:A 6:PEP | 409:A 2:PEP | 429:A 4:PEP | - | - | - | - | - | - | NRLLLTG | CCEECCC | 7 |
| 1EG4 | A | 260 | 82:A 8:PEP | 79:A 11:PEP | 66:A 9:PEP | 1EG3 | A | 260 | 189:A 4:PEP | 80:A 11:PEP | 79:A 11:PEP | NMTPYRSPPPYVP | CCCCCCCCCCCCC | 13 |
| 1ELW | A | 117 | 84:A 2:PEP | 80:A 2:PEP | 49:A 4:PEP | - | - | - | - | - | - | GPTIEEVD | CCCCCCCC | 8 |
| 1ER8 | E | 330 | 12:E 1:PEP | 76:E 4:PEP | 34:E 5:PEP | 1OEW | A | 330 | 221:A 3:PEP | 80:A 2:PEP | 15:A 1:PEP | UPFHLLVY | CCCCCCEC | 8 |
| 1GYB | B | 122 | 45:B 2:PEP | 34:B 4:PEP | 45:B 2:PEP | 1GY7 | B | 122 | 45:B 2:PEP | 36:B 2:PEP | 45:B 2:PEP | GFSFG | CCCCC | 5 |
| 1H6W | A | 151 | 393:A 6:PEP | 362:A 4:PEP | 372:A 8:PEP | - | - | - | - | - | - | SLNYIIKVKE | CCEEEEECCC | 10 |
| 1HC9 | A | 74 | 68:A 3:PEP | 39:A 4:PEP | 9:A 1:PEP | 2ABX | A | 74 | 6:A 3:PEP | 39:A 3:PEP | 74:A 8:PEP | WRYYESSLLPYPD | CEEECCEEEEECC | 13 |
| 1I8K | AB | 225 | 93:A 4:PEP | 356:B 9:PEP | 93:A 4:PEP | - | - | - | - | - | - | KKGNYVVTDH | CECCECCCCC | 10 |
| 1IAK | AB | 367 | 69:A 10:PEP | 85:B 1:PEP | 60:B 12:PEP | - | - | - | - | - | - | STDYGILQINSRW | CECCCCCCCCCCC | 13 |
| 1IHJ | A | 94 | 31:A 1:PEP | 91:A 5:PEP | 91:A 5:PEP | - | - | - | - | - | - | TEFCA | CCCEC | 5 |
| 1JBU | H | 239 | 59:H 15:PEP | 64:H 10:PEP | 251:H 15:PEP | 2BZ6 | H | 254 | 62:H 9:PEP | 254:H 3:PEP | 254:H 3:PEP | EEWEVLCWTWETCER | CCCEEEECCCCCCCC | 15 |
| 1JD5 | A | 105 | 272:A 2:PEP | 286:A 3:PEP | 269:A 4:PEP | 1QBH | A | 103 | 331:A 6:PEP | 313:A 2:PEP | 311:A 3:PEP | AIAYFIPD | CEEEECCC | 8 |
| 1JWG | B | 140 | 95:B 3:PEP | 95:B 3:PEP | 88:B 2:PEP | 1JWF | A | 139 | 92:A 2:PEP | 95:A 3:PEP | 95:A 3:PEP | DLLHI | CCCCC | 5 |
| 1KL3 | C | 120 | 90:C 3:PEP | 79:C 2:PEP | 79:C 2:PEP | 2RTM | A | 123 | 90:A 3:PEP | 47:A 4:PEP | 84:A 2:PEP | HPQFEK | CHHHCC | 6 |
| 1KLU | AB | 369 | 57:B 13:PEP | 78:B 7:PEP | 54:A 4:PEP | 1PYW | AB | 369 | 69:A 11:PEP | 72:A 14:PEP | 54:A 4:PEP | GELIGTLNAAKVPAD | CEECCCCCCCCCCCC | 15 |
| 1LVM | A | 229 | 66:A 7:PEP | 64:A 5:PEP | 68:A 7:PEP | 1LVB | B | 214 | 66:B 7:PEP | 65:B 4:PEP | 67:B 4:PEP | EATQLMN | CCEECCC | 7 |
| 1MFG | A | 95 | 1294:A 8:PEP | 1351:A 7:PEP | 1304:A 2:PEP | 2H3L | A | 103 | 1332:A 9:PEP | 1343:A 5:PEP | 1335:A 6:PEP | EYLGLDVPV | CCCCCCEEC | 9 |
| 1MVU | AB | 333 | 98:A 2:PEP | 52:B 9:PEP | 31:A 3:PEP | - | - | - | - | - | - | VVQEALDKAREGR | CHHHHHHHHHHCC | 13 |
| 1N12 | C | 136 | 137:C 8:PEP | 28:C 10:PEP | 140:C 5:PEP | - | - | - | - | - | - | SDVAFRGNLLD | CEEEEEEEEEC | 11 |
| 1N7F | B | 86 | 682:B 8:PEP | 682:B 8:PEP | 736:B 6:PEP | 1N7E | A | 95 | 685:A 7:PEP | 739:A 8:PEP | 681:A 8:PEP | ATVRTYSC | CCCEEEEC | 8 |
| 1NLN | A | 203 | 110:A 4:PEP | 110:A 4:PEP | 107:A 7:PEP | - | - | - | - | - | - | GVQSLKRRRCF | CCCEEEEEEEC | 11 |
| 1NQ7 | A | 244 | 274:A 7:PEP | 448:A 3:PEP | 444:A 3:PEP | 1N83 | A | 251 | 509:A 3:PEP | 349:A 5:PEP | 352:A 8:PEP | HKILHRLLQE | CHHHHHHHHC | 10 |
| 1NTV | A | 152 | 113:A 4:PEP | 158:A 4:PEP | 114:A 7:PEP | - | - | - | - | - | - | NFDNPVYRKT | CEECCCCCCC | 10 |
| 1NVR | A | 264 | 11:A 2:PEP | 9:A 3:PEP | 10:A 2:PEP | 2QHN | A | 268 | 10:A 2:PEP | 8:A 4:PEP | 11:A 1:PEP | ASVSA | CEEEC | 5 |
| 1NX1 | A | 173 | 125:A 7:PEP | 128:A 7:PEP | 129:A 10:PEP | 1ALV | A | 173 | 125:A 7:PEP | 124:A 3:PEP | 124:A 3:PEP | DAIDALSSDFT | CHHHHHHHHCC | 11 |
| 1OAI | A | 59 | 591:A 5:PEP | 603:A 6:PEP | 613:A 4:PEP | 1GO5 | A | 71 | 54:A 6:PEP | 54:A 6:PEP | 57:A 6:PEP | DSGFSFGSK | CCCCCCCCC | 9 |
| 1OU8 | B | 106 | 58:B 6:PEP | 51:B 2:PEP | 53:B 6:PEP | 1OU9 | A | 121 | 44:A 4:PEP | 53:A 2:PEP | 74:A 8:PEP | GAANDENY | CCCCCCCC | 8 |
| 1PZ5 | AB | 435 | 47:B 3:PEP | 94:A 3:PEP | 93:A 3:PEP | 1M7D | AB | 435 | 97:B 6:PEP | 28:A 7:PEP | 94:A 3:PEP | MDWNMHAA | CCCCCCCC | 8 |
| 1QKZ | H | 219 | 58:H 5:PEP | 33:H 7:PEP | 56:H 3:PEP | - | - | - | - | - | - | ANGGASGQVK | CCCECCECCC | 10 |
| 1RXZ | A | 245 | 220:A 10:PEP | 219:A 10:PEP | 43:A 6:PEP | 1RWZ | A | 244 | 123:A 11:PEP | 151:A 2:PEP | 241:A 3:PEP | KSTQATLERWF | CEEECCHHHCC | 11 |
| 1SE0 | A | 97 | 87:A 3:PEP | 103:A 6:PEP | 86:A 5:PEP | 3SIQ | A | 103 | 77:A 4:PEP | 86:A 4:PEP | 86:A 4:PEP | AIAYFIP | CEEEECC | 7 |
| 1SFI | A | 223 | 41:A 7:PEP | 217:A 2:PEP | 215:A 4:PEP | 1UTN | A | 223 | 215:A 4:PEP | 213:A 5:PEP | 190:A 5:PEP | GRCTKSIPPICFPD | CEEECCCCCCECCC | 14 |
| 1SSH | A | 60 | 38:A 7:PEP | 40:A 9:PEP | 53:A 6:PEP | 1OOT | A | 58 | 51:A 7:PEP | 38:A 10:PEP | 38:A 9:PEP | GPPPAMPARPT | CCCCCCCCCCC | 11 |
| 1SVZ | A | 232 | 181:A 2:PEP | 181:A 2:PEP | 228:A 5:PEP | - | - | - | - | - | - | PQFSLW | CCCCCC | 6 |
| 1T4F | M | 88 | 62:M 3:PEP | 93:M 5:PEP | 62:M 3:PEP | 1Z1M | A | 121 | 96:A 8:PEP | 96:A 9:PEP | 73:A 5:PEP | RFMDYWEGL | CHHHHHCCC | 9 |
| 1T7R | A | 250 | 716:A 8:PEP | 898:A 4:PEP | 894:A 4:PEP | 2AM9 | A | 250 | 716:A 8:PEP | 894:A 3:PEP | 738:A 1:PEP | SSRFESLFAG | CHHHHHHHHC | 10 |
| 1TP5 | A | 115 | 323:A 6:PEP | 376:A 4:PEP | 372:A 2:PEP | 1TQ3 | A | 110 | 322:A 6:PEP | 326:A 3:PEP | 379:A 6:PEP | KKETWV | CCCEEC | 6 |
| 1TW6 | B | 95 | 118:B 6:PEP | 147:B 2:PEP | 121:B 4:PEP | 2I3I | B | 95 | 130:B 4:PEP | 134:B 1:PEP | 147:B 3:PEP | AVPIAQ | CEECCC | 6 |
| 1U00 | A | 227 | 423:A 7:PEP | 434:A 1:PEP | 433:A 4:PEP | - | - | - | - | - | - | ELPPVKIHC | CCCCEECCC | 9 |
| 1U8I | AB | 441 | 52:B 4:PEP | 93:A 2:PEP | 93:A 2:PEP | - | - | - | - | - | - | ELDKWAN | CECCCCC | 7 |
| 1U9L | AB | 138 | 366:A 4:PEP | 369:A 3:PEP | 364:B 4:PEP | - | - | - | - | - | - | NRPILSL | CCCCECC | 7 |
| 1UJ0 | A | 58 | 254:A 2:PEP | 239:A 9:PEP | 216:A 6:PEP | - | - | - | - | - | - | TPMVNRENK | CCCCCHHHC | 9 |
| 1VZQ | H | 250 | 76:H 5:PEP | 76:H 4:PEP | 74:H 2:PEP | 1JWT | A | 299 | 295:A 5:PEP | 105:A 2:PEP | 107:A 6:PEP | DFEEIP | CCCCCC | 6 |
| 1W9E | A | 164 | 258:A 5:PEP | 212:A 4:PEP | 258:A 5:PEP | 1R6J | A | 82 | 258:A 5:PEP | 214:A 2:PEP | 214:A 2:PEP | NEFYF | CCEEC | 5 |
| 1X2R | A | 290 | 334:A 5:PEP | 556:A 5:PEP | 334:A 7:PEP | 1X2J | A | 290 | 602:A 5:PEP | 382:A 7:PEP | 363:A 7:PEP | LDEETGEFL | CCCCCCCCC | 9 |
| 1YMT | A | 235 | 296:A 5:PEP | 296:A 5:PEP | 292:A 9:PEP | - | - | - | - | - | - | RPTILYALLS | CCCHHHHHHC | 10 |
| 1YPH | CE | 228 | 206:E 2:PEP | 122:C 2:PEP | 122:C 1:PEP | - | - | - | - | - | - | CGVPAIQPVL | CCCCCCCCCC | 10 |
| 1YUC | A | 240 | 531:A 7:PEP | 361:A 11:PEP | 375:A 8:PEP | 3TX7 | B | 218 | 374:B 11:PEP | 535:B 7:PEP | 375:B 11:PEP | ASRPAILYALLSSS | CCCCHHHHHHHHCC | 14 |
| 1YWO | A | 55 | 38:A 6:PEP | 11:A 2:PEP | 17:A 8:PEP | 1Y0M | A | 61 | 800:A 3:PEP | 800:A 2:PEP | 800:A 2:PEP | QPPVPPQRPM | CCCCCCCCCC | 10 |
| 1Z9O | AC | 238 | 45:A 7:PEP | 57:A 7:PEP | 45:A 5:PEP | 1Z9L | A | 126 | 45:A 5:PEP | 49:A 7:PEP | 47:A 2:PEP | EDEFYDALS | CCCCEECCC | 9 |
| 1ZUK | AB | 130 | 62:B 6:PEP | 42:B 1:PEP | 41:B 3:PEP | - | - | - | - | - | - | RGPAPPPPPHR | CCCCCCCCCCC | 11 |
| 2A3I | A | 253 | 778:A 9:PEP | 778:A 9:PEP | 799:A 6:PEP | 2AA2 | A | 252 | 962:A 4:PEP | 781:A 9:PEP | 781:A 9:PEP | QQKSLLQQLLTE | CCCCHHHHHHHC | 12 |
| 2AI4 | A | 111 | 37:A 2:PEP | 41:A 8:PEP | 37:A 2:PEP | - | - | - | - | - | - | PQIINRPQ | CEEEECCC | 8 |
| 2AK5 | B | 64 | 43:B 8:PEP | 15:B 2:PEP | 15:B 2:PEP | 2G6F | X | 59 | 58:X 3:PEP | 42:X 6:PEP | 42:X 5:PEP | RPPKPRPR | CCCCCCCC | 8 |
| 2B1Z | B | 238 | 375:B 7:PEP | 358:B 6:PEP | 543:B 3:PEP | 2IOG | A | 243 | 544:A 7:PEP | 358:A 3:PEP | 376:A 3:PEP | KILHRLLQD | CHHHHHHHC | 9 |
| 2B9H | A | 337 | 97:A 11:PEP | 312:A 2:PEP | 313:A 2:PEP | 2B9F | A | 337 | 107:A 9:PEP | 97:A 11:PEP | 314:A 2:PEP | RRNLKGLNLNLH | CCCCCCCCCCCC | 12 |
| 2BBA | A | 185 | 93:A 3:PEP | 100:A 13:PEP | 45:A 5:PEP | - | - | - | - | - | - | NYLFSPNGPIARAW | CCCCCCCCCCHHHC | 14 |
| 2C3I | B | 266 | 239:B 2:PEP | 239:B 2:PEP | 130:B 4:PEP | 2J2I | B | 273 | 204:B 5:PEP | 171:B 4:PEP | 204:B 5:PEP | KRRRHPSG | CCCCCCCC | 8 |
| 2CCH | D | 256 | 214:D 8:PEP | 250:D 10:PEP | 280:D 5:PEP | 1H1R | B | 258 | 285:B 7:PEP | 214:B 8:PEP | 283:B 5:PEP | HTLKGRRLVFDN | CCCCCCCCCCCC | 12 |
| 2D0N | C | 56 | 299:C 7:PEP | 299:C 7:PEP | 297:C 9:PEP | - | - | - | - | - | - | PSIDRSTKP | CCCCHHHCC | 9 |
| 2DS8 | B | 41 | 12:B 5:PEP | 30:B 2:PEP | 12:B 5:PEP | 2DS7 | A | 40 | 27:A 6:PEP | 27:A 6:PEP | 29:A 3:PEP | ALRVVK | CCEECC | 6 |
| 2DZE | AB | 320 | 60:B 5:PEP | 60:B 5:PEP | 72:B 3:PEP | - | - | - | - | - | - | LARRLR | CEEEEC | 6 |
| 2FGR | A | 332 | 7:A 5:PEP | 6:A 3:PEP | 6:A 3:PEP | 2FGQ | X | 330 | 5:X 4:PEP | 285:X 7:PEP | 4:X 3:PEP | DNWQNGTS | CCCECCCC | 8 |
| 2FMF | A | 128 | 90:A 1:PEP | 106:A 13:PEP | 90:A 4:PEP | 1JBE | A | 126 | 92:A 7:PEP | 95:A 13:PEP | 99:A 11:PEP | QDQVDDLLDSLGF | CHHHHHHHHHHCC | 13 |
| 2FNT | AB | 198 | 49:A 3:PEP | 29:A 2:PEP | 45:A 1:PEP | 3HAU | AB | 199 | 45:A 1:PEP | 23:B 4:PEP | 49:B 5:PEP | RQVNFLG | CEECCEC | 7 |
| 2FOJ | A | 137 | 154:A 4:PEP | 118:A 7:PEP | 166:A 4:PEP | 2F1W | A | 144 | 152:A 1:PEP | 154:A 4:PEP | 165:A 6:PEP | GARAHSS | CCCCECC | 7 |
| 2FVJ | A | 258 | 315:A 3:PEP | 314:A 7:PEP | 314:A 7:PEP | 2HWQ | A | 261 | 301:A 7:PEP | 294:A 6:PEP | 471:A 2:PEP | HKLVQLLTTT | CHHHHHHHCC | 10 |
| 2H9M | C | 304 | 107:C 1:PEP | 65:C 3:PEP | 47:C 3:PEP | 2H14 | A | 303 | 89:A 1:PEP | 260:A 5:PEP | 91:A 1:PEP | ARTKQ | CCCCC | 5 |
| 2HO2 | A | 33 | 278:A 6:PEP | 271:A 6:PEP | 269:A 8:PEP | 2E+45 | A | 52 | 38:A 8:PEP | 38:A 9:PEP | 24:A 8:PEP | PPPPPPPPPL | CCCCCCCCCC | 10 |
| 2HPL | A | 100 | 58:A 3:PEP | 34:A 3:PEP | 58:A 3:PEP | 2HPJ | A | 99 | 51:A 4:PEP | 55:A 5:PEP | 60:A 3:PEP | DDLYG | CCCCC | 5 |
| 2IPU | HL | 442 | 93:L 2:PEP | 96:L 3:PEP | 91:L 3:PEP | 1YEJ | HL | 441 | 95:L 3:PEP | 58:H 4:PEP | 96:L 3:PEP | AEFRHDS | CECCCCC | 7 |
| 2IV9 | AB | 469 | 719:B 6:PEP | 819:B 3:PEP | 716:B 6:PEP | - | - | - | - | - | - | SFGDGFADF | CCCCHHHHC | 9 |
| 2J6F | A | 57 | 17:A 3:PEP | 8:A 8:PEP | 17:A 3:PEP | 2J6K | A | 57 | 17:A 3:PEP | 17:A 3:PEP | 37:A 3:PEP | PPKPRPRR | CCCCCCCC | 8 |
| 2JAM | A | 279 | 18:A 2:PEP | 90:A 2:PEP | 88:A 4:PEP | - | - | - | - | - | - | GVSKFA | CCEECC | 6 |
| 2O02 | A | 224 | 211:A 1:PEP | 208:A 1:PEP | 176:A 13:PEP | 3RDH | A | 233 | 213:A 7:PEP | 169:A 10:PEP | 46:A 8:PEP | GHGQGLLDALDLAS | CCCCCHHHHCCCCC | 14 |
| 2O4J | A | 240 | 256:A 3:PEP | 260:A 3:PEP | 242:A 10:PEP | 1IE9 | A | 255 | 416:A 5:PEP | 246:A 10:PEP | 259:A 10:PEP | KNHPMLMNLLKD | CCCHHHHHHHCC | 12 |
| 2O9V | A | 67 | 830:A 1:PEP | 875:A 3:PEP | 855:A 9:PEP | 2O9S | A | 67 | 875:A 3:PEP | 855:A 9:PEP | 858:A 9:PEP | VPPPVPPPPS | CCCCCCCCCC | 10 |
| 2OTU | EF | 233 | 32:E 4:PEP | 32:E 4:PEP | 96:E 5:PEP | - | - | - | - | - | - | QQQQQQQQQQG | CCCCCCCCCEC | 11 |
| 2P0W | A | 319 | 188:A 8:PEP | 276:A 7:PEP | 55:A 13:PEP | - | - | - | - | - | - | KGGKGLGKGGAKRHR | CCCCCCCCCCCCCCC | 15 |
| 2P1K | A | 87 | 70:A 2:PEP | 67:A 5:PEP | 61:A 10:PEP | - | - | - | - | - | - | SATSAKATQTD | CCEEEEEEECC | 11 |
| 2P1T | A | 211 | 302:A 1:PEP | 453:A 1:PEP | 280:A 7:PEP | 3NSQ | A | 193 | 276:A 4:PEP | 276:A 4:PEP | 273:A 3:PEP | HKILHRLLQD | CHHHHHHHHC | 10 |
| 2P54 | A | 267 | 306:A 6:PEP | 462:A 4:PEP | 462:A 4:PEP | 1I7G | A | 259 | 305:A 10:PEP | 462:A 3:PEP | 459:A 5:PEP | ARHKILHRLLQE | CCCHHHHHHHHC | 12 |
| 2PUY | B | 60 | 497:B 7:PEP | 522:B 3:PEP | 489:B 6:PEP | 2YQL | A | 58 | 20:A 3:PEP | 18:A 7:PEP | 21:A 4:PEP | ARTKQTARKS | CCEEEECCCC | 10 |
| 2PV2 | AB | 206 | 240:A 6:PEP | 185:A 12:PEP | 263:A 10:PEP | - | - | - | - | - | - | NFTLKFWDIFRK | CCHHHHHHHHCC | 12 |
| 2QOS | C | 173 | 77:C 10:PEP | 40:C 7:PEP | 120:C 5:PEP | 1LF7 | A | 164 | 120:A 6:PEP | 122:A 4:PEP | 122:A 4:PEP | LRYDSTAERLY | CCEECCCCEEC | 11 |
| 2R7G | C | 337 | 646:C 8:PEP | 476:C 8:PEP | 476:C 8:PEP | 3POM | A | 342 | 530:A 7:PEP | 530:A 7:PEP | 464:A 4:PEP | PPTLHELYDL | CCCHHHHCCC | 10 |
| 2V3S | B | 96 | 473:B 3:PEP | 448:B 5:PEP | 473:B 3:PEP | - | - | - | - | - | - | GRFQVT | CCCEEC | 6 |
| 2VJ0 | A | 246 | 715:A 7:PEP | 715:A 7:PEP | 715:A 7:PEP | 1B9K | A | 237 | 726:A 7:PEP | 715:A 7:PEP | 742:A 4:PEP | PKGWVTFE | CCCCCCCC | 8 |
| 2ZJD | A | 121 | 66:A 10:PEP | 53:A 6:PEP | 53:A 8:PEP | 1V49 | A | 120 | 49:A 7:PEP | 53:A 8:PEP | 49:A 7:PEP | GGDDDWTHLS | CCCCCCEECC | 10 |
| 3BFQ | G | 132 | 21:G 3:PEP | 86:G 7:PEP | 23:G 5:PEP | - | - | - | - | - | - | ADSTITIRGYVRDNR | CCEEEEEEEEEECCC | 15 |
| 3BU3 | A | 297 | 1086:A 2:PEP | 1003:A 3:PEP | 1173:A 11:PEP | 3EKK | A | 285 | 1005:A 1:PEP | 1215:A 9:PEP | 1184:A 14:PEP | YNPYPEDYGDIEIG | CCCCHHHCEEEEEC | 14 |
| 3BWA | A | 276 | 76:A 7:PEP | 167:A 1:PEP | 167:A 1:PEP | - | - | - | - | - | - | FPTKDVAL | CCCCCCCC | 8 |
| 3CVP | A | 279 | 429:A 5:PEP | 429:A 6:PEP | 569:A 5:PEP | - | - | - | - | - | - | NRASKL | CCCCCC | 6 |
| 3D1E | A | 366 | 320:A 2:PEP | 346:A 5:PEP | 175:A 4:PEP | 3D1G | A | 366 | 175:A 4:PEP | 362:A 3:PEP | 362:A 4:PEP | GQLGLF | CECCCC | 6 |
| 3D9T | B | 95 | 323:B 3:PEP | 307:B 3:PEP | 307:B 1:PEP | 2QBH | A | 103 | 325:A 1:PEP | 316:A 1:PEP | 315:A 1:PEP | ATPFQE | CEEECC | 6 |

**Table S2. BENCHMARK RESULTS – all bound targets.** CABS-dock performance for 101 bound cases (listed in rows) in 3 independent prediction runs (shown in separate columns). The table shows the lowest peptide-RMSD values (calculated on the peptide only after superimposition of the receptor structures) among: all 10,000 models (all), top 1000 models (top 1k, selected during filtering and clustering procedure), top 100 models (top 100, selected through further clustering) and top 10 final models (top 10). Fourth column shows the lowest RMSD values obtained in three prediction runs. For comparison purposes, the last column contains lowest RMSD values obtained in three prediction runs without contact information.

|  | contact info (run 1) | | | | contact info (run 2) | | | | contact info (run 3) | | | | contact info (best result) | | | | no contact info (best result) | | | |
| --- | --- | --- | --- | --- | --- | --- | --- | --- | --- | --- | --- | --- | --- | --- | --- | --- | --- | --- | --- | --- |
|  | **10k** | **1k** | **100** | **10** | **10k** | **1k** | **100** | **10** | **10k** | **1k** | **100** | **10** | **10k** | **1k** | **100** | **10** | **10k** | **1k** | **100** | **10** |
| 1AWR | 1.30 | 1.30 | 2.35 | 2.82 | 1.54 | 2.37 | 2.91 | 3.33 | 1.43 | 1.43 | 2.58 | 2.98 | **1.30** | **1.30** | **2.35** | **2.82** | 1.51 | 1.89 | 2.82 | 2.83 |
| 1CE1 | 1.72 | 2.19 | 2.38 | 2.91 | 1.52 | 1.83 | 3.43 | 3.72 | 2.06 | 2.76 | 2.84 | 3.12 | **1.52** | **1.83** | **2.38** | **2.91** | 1.73 | 2.14 | 3.34 | 3.86 |
| 1CKA | 2.50 | 2.50 | 3.96 | 4.09 | 2.77 | 2.92 | 4.17 | 4.15 | 2.80 | 3.05 | 3.24 | 3.71 | **2.50** | **2.50** | **3.24** | **3.71** | 3.09 | 3.37 | 4.03 | 4.45 |
| 1CZY | 1.63 | 1.88 | 2.03 | 2.52 | 1.83 | 1.83 | 1.83 | 2.01 | 1.81 | 1.81 | 1.82 | 2.19 | **1.63** | **1.81** | **1.82** | **2.01** | 1.91 | 2.21 | 2.40 | 3.05 |
| 1D4T | 1.27 | 1.27 | 1.68 | 2.28 | 1.38 | 1.38 | 1.89 | 2.28 | 1.82 | 2.22 | 2.45 | 2.38 | **1.27** | **1.27** | **1.68** | **2.28** | 1.97 | 1.97 | 2.20 | 2.63 |
| 1DDV | 2.49 | 2.75 | 3.44 | 4.67 | 1.97 | 1.97 | 3.62 | 3.81 | 2.37 | 2.44 | 4.08 | 4.52 | **1.97** | **1.97** | **3.44** | **3.81** | 2.52 | 2.94 | 3.97 | 3.82 |
| 1DKX | 7.92 | 8.36 | 8.87 | 10.18 | 6.75 | 10.18 | 11.41 | 12.22 | 8.23 | 8.69 | 8.93 | 9.31 | **6.75** | **8.36** | **8.87** | **9.31** | 7.93 | 7.93 | 8.19 | 8.84 |
| 1EG4 | 4.58 | 10.95 | 12.57 | 13.53 | 4.52 | 5.48 | 7.78 | 9.45 | 4.03 | 6.12 | 6.72 | 9.36 | **4.03** | **5.48** | **6.72** | **9.36** | 6.57 | 8.61 | 8.97 | 11.08 |
| 1ELW | 1.45 | 2.21 | 2.30 | 2.82 | 1.75 | 1.75 | 3.35 | 3.32 | 1.81 | 1.81 | 2.46 | 3.04 | **1.45** | **1.75** | **2.30** | **2.82** | 1.96 | 1.96 | 2.53 | 3.80 |
| 1ER8 | 1.69 | 1.69 | 2.60 | 2.24 | 6.99 | 6.99 | 7.62 | 7.56 | 6.07 | 6.07 | 6.68 | 13.38 | **1.69** | **1.69** | **2.60** | **2.24** | 6.33 | 6.39 | 6.90 | 6.92 |
| 1GYB | 2.05 | 2.05 | 3.92 | 4.36 | 2.29 | 3.24 | 5.48 | 9.95 | 2.32 | 2.32 | 4.52 | 3.57 | **2.05** | **2.05** | **3.92** | **3.57** | 4.53 | 7.50 | 10.99 | 14.83 |
| 1H6W | 6.48 | 7.77 | 9.45 | 15.97 | 8.24 | 8.26 | 8.90 | 9.38 | 7.52 | 10.55 | 12.95 | 18.63 | **6.48** | **7.77** | **8.90** | **9.38** | 6.10 | 8.22 | 12.11 | 20.32 |
| 1HC9 | 5.04 | 6.47 | 8.07 | 8.71 | 5.09 | 5.62 | 6.21 | 6.71 | 5.51 | 5.99 | 7.03 | 7.41 | **5.04** | **5.62** | **6.21** | **6.71** | 6.21 | 6.52 | 7.17 | 7.94 |
| 1I8K | 2.42 | 2.42 | 2.42 | 3.19 | 3.17 | 3.20 | 3.71 | 4.67 | 2.08 | 2.32 | 2.32 | 2.53 | **2.08** | **2.32** | **2.32** | **2.53** | 3.60 | 4.00 | 4.04 | 7.64 |
| 1IAK | 2.59 | 2.70 | 2.88 | 3.82 | 2.66 | 3.02 | 3.68 | 3.68 | 2.47 | 2.58 | 2.91 | 3.52 | **2.47** | **2.58** | **2.88** | **3.52** | 3.40 | 3.98 | 4.19 | 5.13 |
| 1IHJ | 1.81 | 2.87 | 2.91 | 4.26 | 1.53 | 1.53 | 3.26 | 3.78 | 1.73 | 1.73 | 3.42 | 3.92 | **1.53** | **1.53** | **2.91** | **3.78** | 2.17 | 2.72 | 4.42 | 6.50 |
| 1JBU | 6.38 | 7.21 | 9.33 | 10.95 | 4.41 | 5.65 | 6.57 | 8.79 | 5.63 | 6.00 | 6.00 | 12.31 | **4.41** | **5.65** | **6.00** | **8.79** | 7.39 | 9.67 | 13.45 | 19.68 |
| 1JD5 | 2.30 | 2.30 | 3.24 | 3.42 | 2.71 | 3.13 | 4.56 | 5.61 | 2.29 | 2.83 | 5.28 | 5.63 | **2.29** | **2.30** | **3.24** | **3.42** | 2.85 | 2.85 | 5.88 | 15.18 |
| 1JWG | 1.86 | 2.06 | 2.06 | 4.28 | 1.80 | 2.24 | 4.13 | 4.66 | 2.15 | 2.15 | 2.95 | 4.28 | **1.80** | **2.06** | **2.06** | **4.28** | 2.29 | 2.29 | 3.92 | 5.27 |
| 1KL3 | 2.63 | 3.33 | 4.47 | 6.02 | 1.92 | 3.87 | 4.87 | 4.86 | 1.88 | 3.10 | 4.80 | 5.16 | **1.88** | **3.10** | **4.47** | **4.86** | 2.45 | 3.51 | 4.82 | 5.21 |
| 1KLU | 3.18 | 3.82 | 3.89 | 4.64 | 3.08 | 3.22 | 4.14 | 4.64 | 3.17 | 3.48 | 3.92 | 4.41 | **3.08** | **3.22** | **3.89** | **4.41** | 3.57 | 3.80 | 4.50 | 5.30 |
| 1LVM | 1.47 | 1.47 | 2.30 | 2.41 | 1.64 | 1.95 | 2.12 | 2.59 | 1.96 | 2.57 | 7.28 | 6.16 | **1.47** | **1.47** | **2.12** | **2.41** | 5.42 | 6.07 | 12.52 | 13.47 |
| 1MFG | 2.98 | 3.20 | 3.99 | 5.04 | 3.37 | 3.37 | 4.78 | 6.42 | 3.22 | 3.81 | 5.55 | 6.06 | **2.98** | **3.20** | **3.99** | **5.04** | 2.99 | 3.71 | 6.78 | 6.78 |
| 1MVU | 2.91 | 3.72 | 4.08 | 5.22 | 2.94 | 3.23 | 4.34 | 4.06 | 2.64 | 2.89 | 3.53 | 3.55 | **2.64** | **2.89** | **3.53** | **3.55** | 3.29 | 4.26 | 4.89 | 5.29 |
| 1N12 | 1.52 | 2.14 | 2.16 | 3.08 | 8.72 | 11.37 | 13.66 | 14.51 | 2.86 | 3.69 | 3.87 | 4.43 | **1.52** | **2.14** | **2.16** | **3.08** | 8.31 | 8.31 | 8.72 | 10.46 |
| 1N7F | 2.40 | 2.98 | 2.98 | 5.94 | 2.09 | 2.09 | 3.62 | 4.64 | 1.78 | 2.32 | 4.52 | 4.79 | **1.78** | **2.09** | **2.98** | **4.64** | 4.18 | 6.20 | 7.81 | 9.15 |
| 1NLN | 3.10 | 4.84 | 6.28 | 7.07 | 3.35 | 3.50 | 3.87 | 4.23 | 4.79 | 4.79 | 5.15 | 7.24 | **3.10** | **3.50** | **3.87** | **4.23** | 3.07 | 3.62 | 3.74 | 5.23 |
| 1NQ7 | 0.86 | 0.92 | 1.08 | 1.86 | 0.96 | 1.27 | 1.51 | 1.83 | 0.96 | 1.01 | 1.05 | 1.01 | **0.86** | **0.92** | **1.05** | **1.01** | 1.03 | 1.03 | 1.11 | 2.73 |
| 1NTV | 2.61 | 3.12 | 3.63 | 3.86 | 2.77 | 2.79 | 3.08 | 4.07 | 1.90 | 2.32 | 2.73 | 2.97 | **1.90** | **2.32** | **2.73** | **2.97** | 2.89 | 2.89 | 3.71 | 5.28 |
| 1NVR | 2.14 | 3.34 | 3.41 | 6.33 | 1.19 | 1.19 | 1.34 | 3.84 | 1.28 | 1.66 | 3.30 | 3.58 | **1.19** | **1.19** | **1.34** | **3.58** | 1.68 | 1.68 | 4.06 | 4.15 |
| 1NX1 | 1.33 | 2.12 | 2.50 | 2.37 | 1.56 | 1.56 | 1.56 | 3.70 | 1.96 | 1.96 | 2.26 | 2.71 | **1.33** | **1.56** | **1.56** | **2.37** | 2.82 | 2.94 | 3.26 | 4.20 |
| 1OAI | 2.53 | 3.47 | 5.38 | 5.12 | 3.36 | 3.36 | 4.48 | 5.11 | 3.01 | 3.35 | 4.24 | 6.25 | **2.53** | **3.35** | **4.24** | **5.11** | 3.04 | 3.56 | 5.27 | 6.53 |
| 1OU8 | 2.52 | 3.32 | 4.50 | 5.73 | 3.08 | 3.19 | 3.64 | 5.13 | 3.65 | 3.74 | 5.54 | 5.13 | **2.52** | **3.19** | **3.64** | **5.13** | 2.98 | 3.97 | 5.65 | 5.56 |
| 1PZ5 | 2.24 | 2.99 | 3.04 | 3.03 | 2.25 | 2.52 | 2.61 | 3.19 | 2.13 | 2.13 | 3.11 | 2.88 | **2.13** | **2.13** | **2.61** | **2.88** | 4.58 | 4.58 | 5.32 | 5.22 |
| 1QKZ | 4.62 | 5.12 | 6.43 | 7.39 | 3.61 | 4.06 | 5.86 | 6.75 | 4.03 | 4.44 | 6.15 | 8.26 | **3.61** | **4.06** | **5.86** | **6.75** | 5.00 | 6.45 | 6.96 | 10.69 |
| 1RXZ | 2.29 | 4.05 | 4.29 | 6.09 | 3.34 | 3.95 | 5.66 | 6.31 | 3.24 | 3.53 | 4.28 | 5.55 | **2.29** | **3.53** | **4.28** | **5.55** | 3.54 | 3.90 | 6.16 | 6.11 |
| 1SE0 | 3.33 | 3.63 | 4.74 | 5.48 | 3.01 | 3.74 | 4.85 | 4.97 | 3.37 | 4.14 | 5.27 | 4.71 | **3.01** | **3.63** | **4.74** | **4.71** | 4.05 | 4.05 | 9.00 | 7.55 |
| 1SFI | 4.94 | 5.54 | 6.02 | 6.43 | 4.22 | 4.59 | 4.59 | 5.15 | 4.26 | 4.47 | 4.60 | 6.31 | **4.22** | **4.47** | **4.59** | **5.15** | 6.74 | 7.18 | 7.36 | 7.77 |
| 1SSH | 3.73 | 4.22 | 5.31 | 6.33 | 3.12 | 3.12 | 4.78 | 5.96 | 3.40 | 3.95 | 5.51 | 6.09 | **3.12** | **3.12** | **4.78** | **5.96** | 3.00 | 4.32 | 5.33 | 5.41 |
| 1SVZ | 1.28 | 1.48 | 1.92 | 2.90 | 1.77 | 1.77 | 1.77 | 2.41 | 1.99 | 2.52 | 2.73 | 3.25 | **1.28** | **1.48** | **1.77** | **2.41** | 2.17 | 2.17 | 3.77 | 5.13 |
| 1T4F | 2.05 | 2.40 | 3.29 | 3.64 | 2.34 | 2.34 | 3.20 | 3.90 | 2.19 | 2.26 | 2.71 | 2.92 | **2.05** | **2.26** | **2.71** | **2.92** | 2.59 | 2.79 | 2.93 | 3.02 |
| 1T7R | 1.02 | 1.65 | 1.65 | 2.63 | 1.05 | 1.39 | 1.44 | 1.63 | 0.97 | 1.37 | 1.73 | 2.40 | **0.97** | **1.37** | **1.44** | **1.63** | 1.61 | 1.92 | 2.05 | 1.88 |
| 1TP5 | 0.95 | 1.13 | 1.46 | 1.69 | 1.14 | 1.16 | 1.26 | 2.33 | 1.23 | 1.23 | 1.49 | 1.95 | **0.95** | **1.13** | **1.26** | **1.69** | 1.15 | 1.45 | 2.46 | 3.57 |
| 1TW6 | 3.12 | 3.12 | 3.12 | 9.24 | 2.18 | 2.18 | 2.70 | 2.99 | 2.50 | 3.63 | 4.90 | 6.90 | **2.18** | **2.18** | **2.70** | **2.99** | 3.27 | 3.93 | 10.22 | 7.22 |
| 1U00 | 9.14 | 9.14 | 10.91 | 12.00 | 11.24 | 12.39 | 13.78 | 13.95 | 7.82 | 12.45 | 13.01 | 12.80 | **7.82** | **9.14** | **10.91** | **12.00** | 8.89 | 8.89 | 9.28 | 11.21 |
| 1U8I | 2.81 | 2.81 | 3.85 | 3.50 | 2.30 | 2.80 | 2.80 | 5.41 | 2.74 | 2.84 | 3.53 | 3.84 | **2.30** | **2.80** | **2.80** | **3.50** | 5.75 | 9.66 | 10.10 | 13.84 |
| 1U9L | 2.97 | 2.97 | 5.27 | 5.42 | 2.82 | 3.96 | 4.61 | 5.41 | 3.73 | 3.73 | 3.73 | 4.82 | **2.82** | **2.97** | **3.73** | **4.82** | 8.77 | 8.77 | 8.96 | 9.25 |
| 1UJ0 | 2.84 | 2.92 | 3.73 | 2.95 | 2.76 | 3.28 | 3.85 | 4.68 | 2.68 | 3.56 | 4.42 | 4.47 | **2.68** | **2.92** | **3.73** | **2.95** | 3.44 | 3.49 | 4.29 | 4.83 |
| 1VZQ | 1.69 | 2.13 | 2.84 | 3.27 | 1.84 | 2.18 | 2.18 | 3.03 | 2.16 | 2.20 | 2.74 | 2.81 | **1.69** | **2.13** | **2.18** | **2.81** | 2.76 | 4.02 | 5.03 | 6.71 |
| 1W9E | 0.99 | 1.05 | 1.93 | 2.17 | 0.69 | 0.69 | 0.77 | 1.47 | 1.54 | 1.54 | 2.47 | 4.02 | **0.69** | **0.69** | **0.77** | **1.47** | 3.77 | 5.76 | 9.04 | 16.41 |
| 1X2R | 3.54 | 4.21 | 5.19 | 6.87 | 3.26 | 3.72 | 3.77 | 3.91 | 3.49 | 4.10 | 4.63 | 5.93 | **3.26** | **3.72** | **3.77** | **3.91** | 2.89 | 4.85 | 5.04 | 5.10 |
| 1YMT | 2.24 | 2.42 | 3.19 | 3.20 | 1.46 | 1.51 | 2.74 | 3.17 | 2.28 | 2.54 | 2.54 | 3.95 | **1.46** | **1.51** | **2.54** | **3.17** | 2.54 | 2.77 | 2.89 | 3.28 |
| 1YPH | 3.30 | 3.44 | 3.73 | 4.36 | 3.20 | 3.71 | 4.35 | 4.89 | 3.01 | 3.09 | 4.63 | 4.65 | **3.01** | **3.09** | **3.73** | **4.36** | 3.27 | 4.03 | 4.66 | 4.63 |
| 1YUC | 1.49 | 2.07 | 3.07 | 2.80 | 1.23 | 1.63 | 1.87 | 2.16 | 2.02 | 2.36 | 2.58 | 2.73 | **1.23** | **1.63** | **1.87** | **2.16** | 2.89 | 3.68 | 5.25 | 6.13 |
| 1YWO | 3.08 | 3.37 | 4.05 | 3.87 | 2.48 | 3.16 | 4.58 | 4.75 | 3.29 | 4.19 | 5.17 | 5.32 | **2.48** | **3.16** | **4.05** | **3.87** | 2.97 | 4.25 | 5.98 | 6.46 |
| 1Z9O | 3.67 | 5.45 | 7.44 | 8.08 | 3.39 | 4.95 | 6.44 | 7.71 | 3.88 | 6.04 | 8.41 | 9.48 | **3.39** | **4.95** | **6.44** | **7.71** | 4.61 | 6.03 | 6.83 | 8.28 |
| 1ZUK | 2.87 | 3.41 | 4.08 | 3.99 | 2.83 | 3.40 | 4.10 | 4.59 | 2.85 | 3.26 | 3.55 | 3.80 | **2.83** | **3.26** | **3.55** | **3.80** | 2.93 | 3.18 | 3.64 | 4.43 |
| 2A3I | 1.42 | 1.42 | 2.52 | 3.39 | 1.74 | 1.74 | 3.06 | 2.79 | 2.00 | 2.12 | 2.84 | 4.30 | **1.42** | **1.42** | **2.52** | **2.79** | 1.68 | 2.10 | 2.10 | 4.02 |
| 2AI4 | 1.96 | 2.32 | 3.10 | 4.59 | 3.86 | 4.50 | 4.54 | 4.75 | 2.08 | 3.21 | 4.36 | 6.51 | **1.96** | **2.32** | **3.10** | **4.59** | 5.94 | 8.48 | 8.51 | 9.49 |
| 2AK5 | 2.80 | 3.72 | 4.79 | 5.98 | 2.67 | 3.01 | 3.31 | 3.82 | 2.40 | 2.77 | 3.80 | 4.31 | **2.40** | **2.77** | **3.31** | **3.82** | 3.57 | 3.57 | 4.79 | 5.38 |
| 2B1Z | 0.71 | 0.77 | 1.24 | 1.54 | 0.90 | 1.24 | 1.66 | 1.10 | 0.86 | 0.86 | 1.18 | 1.28 | **0.71** | **0.77** | **1.18** | **1.10** | 0.82 | 1.05 | 1.18 | 1.16 |
| 2B9H | 2.16 | 2.37 | 4.01 | 4.10 | 2.70 | 2.70 | 2.98 | 3.95 | 2.57 | 3.21 | 9.87 | 8.70 | **2.16** | **2.37** | **2.98** | **3.95** | 2.57 | 2.90 | 3.11 | 2.96 |
| 2BBA | 3.87 | 3.87 | 4.66 | 6.64 | 2.98 | 3.37 | 3.95 | 4.57 | 3.68 | 4.09 | 5.17 | 5.50 | **2.98** | **3.37** | **3.95** | **4.57** | 3.53 | 3.65 | 5.17 | 5.26 |
| 2C3I | 2.69 | 2.69 | 3.56 | 4.67 | 2.23 | 2.23 | 3.28 | 4.45 | 3.33 | 3.65 | 8.41 | 10.21 | **2.23** | **2.23** | **3.28** | **4.45** | 3.16 | 6.05 | 8.61 | 11.16 |
| 2CCH | 3.40 | 4.23 | 5.14 | 7.02 | 3.73 | 3.94 | 5.44 | 7.65 | 2.85 | 5.97 | 7.36 | 10.54 | **2.85** | **3.94** | **5.14** | **7.02** | 3.36 | 5.26 | 10.11 | 11.47 |
| 2D0N | 2.66 | 2.66 | 3.19 | 4.28 | 2.62 | 2.62 | 2.89 | 4.03 | 2.61 | 2.61 | 2.61 | 4.70 | **2.61** | **2.61** | **2.61** | **4.03** | 2.64 | 2.64 | 4.22 | 4.68 |
| 2DS8 | 0.69 | 1.03 | 1.08 | 1.26 | 2.91 | 6.33 | 9.75 | 11.43 | 0.70 | 1.06 | 1.10 | 2.32 | **0.69** | **1.03** | **1.08** | **1.26** | 2.15 | 2.15 | 5.45 | 9.42 |
| 2DZE | 3.63 | 4.27 | 4.36 | 5.12 | 0.87 | 0.87 | 0.95 | 1.33 | 0.70 | 0.70 | 1.13 | 1.34 | **0.70** | **0.70** | **0.95** | **1.33** | 2.39 | 2.71 | 2.71 | 3.13 |
| 2FGR | 6.30 | 12.39 | 13.08 | 14.73 | 4.55 | 7.58 | 8.52 | 10.75 | 4.76 | 6.82 | 9.80 | 8.85 | **4.55** | **6.82** | **8.52** | **8.85** | 4.96 | 13.42 | 13.75 | 15.87 |
| 2FMF | 3.95 | 4.24 | 4.73 | 5.64 | 3.30 | 4.66 | 5.40 | 5.82 | 3.03 | 4.27 | 4.92 | 5.71 | **3.03** | **4.24** | **4.73** | **5.64** | 2.87 | 2.87 | 5.92 | 6.43 |
| 2FNT | 0.82 | 0.95 | 1.08 | 1.35 | 0.98 | 1.17 | 1.24 | 1.22 | 4.05 | 4.06 | 4.15 | 4.16 | **0.82** | **0.95** | **1.08** | **1.22** | 0.76 | 0.80 | 0.80 | 1.12 |
| 2FOJ | 2.14 | 2.83 | 3.58 | 5.59 | 2.83 | 3.08 | 3.35 | 7.19 | 2.52 | 2.91 | 3.96 | 5.41 | **2.14** | **2.83** | **3.35** | **5.41** | 2.65 | 2.92 | 3.94 | 4.40 |
| 2FVJ | 1.15 | 1.26 | 1.39 | 1.33 | 0.95 | 1.19 | 1.29 | 1.32 | 0.97 | 1.27 | 1.33 | 1.35 | **0.95** | **1.19** | **1.29** | **1.32** | 1.46 | 1.46 | 1.46 | 2.43 |
| 2H9M | 1.44 | 1.65 | 1.71 | 2.08 | 1.63 | 2.19 | 2.73 | 3.27 | 1.69 | 1.69 | 2.98 | 4.20 | **1.44** | **1.65** | **1.71** | **2.08** | 1.36 | 1.80 | 1.80 | 4.42 |
| 2HO2 | 3.41 | 4.21 | 4.94 | 6.48 | 3.43 | 4.80 | 6.14 | 5.58 | 3.72 | 4.34 | 4.59 | 5.23 | **3.41** | **4.21** | **4.59** | **5.23** | 3.80 | 4.89 | 6.33 | 5.59 |
| 2HPL | 1.74 | 1.74 | 1.74 | 2.79 | 1.38 | 1.73 | 2.22 | 4.12 | 1.63 | 1.63 | 3.34 | 4.63 | **1.38** | **1.63** | **1.74** | **2.79** | 1.28 | 2.08 | 3.45 | 3.94 |
| 2IPU | 1.65 | 1.74 | 1.88 | 2.32 | 1.31 | 1.57 | 2.60 | 2.69 | 1.76 | 2.08 | 2.35 | 3.96 | **1.31** | **1.57** | **1.88** | **2.32** | 5.04 | 5.50 | 6.47 | 8.75 |
| 2IV9 | 2.67 | 2.73 | 2.87 | 4.38 | 3.03 | 3.30 | 4.67 | 5.00 | 2.86 | 2.86 | 3.78 | 4.19 | **2.67** | **2.73** | **2.87** | **4.19** | 3.06 | 4.10 | 6.59 | 6.73 |
| 2J6F | 2.98 | 2.98 | 3.04 | 3.93 | 2.69 | 2.89 | 2.96 | 3.09 | 2.68 | 2.68 | 3.42 | 6.99 | **2.68** | **2.68** | **2.96** | **3.09** | 2.96 | 3.49 | 3.95 | 5.29 |
| 2JAM | 1.95 | 1.95 | 3.21 | 5.81 | 2.65 | 3.69 | 6.59 | 7.67 | 2.02 | 2.02 | 4.34 | 8.64 | **1.95** | **1.95** | **3.21** | **5.81** | 4.34 | 7.51 | 11.81 | 13.62 |
| 2O02 | 3.49 | 6.16 | 7.83 | 14.15 | 3.02 | 3.19 | 4.22 | 5.41 | 3.61 | 3.68 | 4.45 | 5.98 | **3.02** | **3.19** | **4.22** | **5.41** | 3.54 | 3.71 | 4.00 | 4.92 |
| 2O4J | 1.17 | 1.51 | 1.51 | 2.23 | 1.46 | 1.64 | 1.84 | 1.91 | 1.03 | 1.63 | 1.97 | 1.82 | **1.03** | **1.51** | **1.51** | **1.82** | 1.49 | 2.02 | 2.41 | 2.81 |
| 2O9V | 3.00 | 3.35 | 3.91 | 3.74 | 2.85 | 2.85 | 4.05 | 5.37 | 2.69 | 2.69 | 4.20 | 4.26 | **2.69** | **2.69** | **3.91** | **3.74** | 3.38 | 3.73 | 4.60 | 5.39 |
| 2OTU | 3.73 | 4.25 | 4.50 | 6.47 | 3.12 | 3.81 | 4.96 | 4.75 | 3.48 | 3.68 | 4.10 | 4.75 | **3.12** | **3.68** | **4.10** | **4.75** | 3.15 | 4.24 | 6.09 | 8.79 |
| 2P0W | 4.69 | 4.82 | 5.73 | 7.16 | 4.74 | 5.23 | 6.04 | 7.05 | 4.25 | 4.31 | 6.15 | 7.14 | **4.25** | **4.31** | **5.73** | **7.05** | 5.88 | 6.26 | 6.38 | 14.35 |
| 2P1K | 2.04 | 2.45 | 3.07 | 4.03 | 2.43 | 2.44 | 2.66 | 3.50 | 2.56 | 2.82 | 3.64 | 5.55 | **2.04** | **2.44** | **2.66** | **3.50** | 2.47 | 2.47 | 3.06 | 4.08 |
| 2P1T | 0.77 | 0.77 | 0.77 | 0.77 | 0.61 | 0.80 | 0.81 | 0.91 | 0.84 | 1.37 | 1.37 | 1.23 | **0.61** | **0.77** | **0.77** | **0.77** | 0.90 | 0.90 | 1.30 | 1.37 |
| 2P54 | 1.69 | 2.66 | 3.23 | 4.03 | 1.76 | 2.56 | 2.57 | 3.69 | 1.82 | 1.83 | 2.59 | 2.62 | **1.69** | **1.83** | **2.57** | **2.62** | 1.66 | 2.25 | 2.61 | 2.02 |
| 2PUY | 2.49 | 2.49 | 2.94 | 3.38 | 3.98 | 3.98 | 6.70 | 8.69 | 1.78 | 1.94 | 3.87 | 3.51 | **1.78** | **1.94** | **2.94** | **3.38** | 3.63 | 3.63 | 8.70 | 10.35 |
| 2PV2 | 1.87 | 2.06 | 2.45 | 2.23 | 1.56 | 1.56 | 1.87 | 2.05 | 2.13 | 2.17 | 2.33 | 2.38 | **1.56** | **1.56** | **1.87** | **2.05** | 1.72 | 1.72 | 1.72 | 3.77 |
| 2QOS | 3.10 | 3.40 | 3.87 | 3.84 | 2.89 | 3.22 | 5.03 | 6.18 | 2.64 | 2.64 | 3.35 | 4.45 | **2.64** | **2.64** | **3.35** | **3.84** | 3.11 | 3.94 | 4.65 | 5.76 |
| 2R7G | 2.80 | 3.14 | 4.09 | 3.98 | 1.80 | 1.80 | 4.37 | 3.44 | 1.56 | 2.35 | 2.46 | 4.42 | **1.56** | **1.80** | **2.46** | **3.44** | 1.47 | 1.62 | 1.62 | 3.33 |
| 2V3S | 0.77 | 0.77 | 0.77 | 2.12 | 0.96 | 0.96 | 0.98 | 1.28 | 0.82 | 1.21 | 1.21 | 1.55 | **0.77** | **0.77** | **0.77** | **1.28** | 1.05 | 1.09 | 1.74 | 2.00 |
| 2VJ0 | 2.33 | 2.88 | 3.98 | 3.79 | 2.59 | 2.91 | 5.28 | 6.58 | 2.54 | 2.85 | 4.04 | 4.85 | **2.33** | **2.85** | **3.98** | **3.79** | 2.09 | 2.96 | 4.12 | 3.91 |
| 2ZJD | 1.34 | 1.69 | 1.87 | 2.63 | 1.52 | 1.95 | 2.62 | 2.72 | 1.61 | 1.61 | 2.02 | 3.38 | **1.34** | **1.61** | **1.87** | **2.63** | 1.57 | 1.58 | 1.91 | 3.70 |
| 3BFQ | 1.55 | 1.59 | 1.68 | 2.28 | 1.57 | 1.62 | 2.01 | 1.85 | 1.29 | 1.33 | 2.15 | 3.40 | **1.29** | **1.33** | **1.68** | **1.85** | 1.24 | 1.41 | 1.83 | 2.58 |
| 3BU3 | 2.61 | 3.07 | 6.22 | 3.96 | 5.28 | 5.96 | 8.11 | 9.26 | 2.94 | 3.54 | 3.84 | 3.82 | **2.61** | **3.07** | **3.84** | **3.82** | 5.77 | 5.77 | 7.71 | 8.86 |
| 3BWA | 1.90 | 2.19 | 2.44 | 3.37 | 2.15 | 2.15 | 2.66 | 2.87 | 2.09 | 2.20 | 2.34 | 2.46 | **1.90** | **2.15** | **2.34** | **2.46** | 1.83 | 2.51 | 2.98 | 3.20 |
| 3CVP | 3.29 | 3.51 | 4.39 | 5.95 | 2.26 | 2.76 | 3.95 | 5.94 | 2.78 | 2.78 | 2.78 | 4.85 | **2.26** | **2.76** | **2.78** | **4.85** | 3.07 | 4.25 | 5.49 | 7.59 |
| 3D1E | 1.51 | 2.26 | 2.36 | 5.56 | 2.28 | 2.28 | 4.58 | 5.12 | 2.67 | 2.67 | 4.65 | 9.48 | **1.51** | **2.26** | **2.36** | **5.12** | 2.57 | 2.57 | 8.18 | 8.93 |
| 3D9T | 2.28 | 2.90 | 3.17 | 3.66 | 2.09 | 2.62 | 3.43 | 3.77 | 1.84 | 2.19 | 2.66 | 3.35 | **1.84** | **2.19** | **2.66** | **3.35** | 3.02 | 3.91 | 7.25 | 9.16 |
| **MEAN** | **2.62** | **3.16** | **3.85** | **4.79** | **2.74** | **3.20** | **4.10** | **4.85** | **2.70** | **3.14** | **4.10** | **5.02** | **2.29** | **2.66** | **3.25** | **3.93** | **3.33** | **4.05** | **5.36** | **6.64** |

**Table S3. BENCHMARK RESULTS – all unbound targets.** CABS-dock performance for 68 unbound cases (listed in rows) in 3 independent prediction runs (shown in separate columns). The table shows the lowest peptide-RMSD values (calculated on the peptide only after superimposition of the receptor structures) among: all 10,000 models (all), top 1000 models (top 1k, selected during filtering and clustering procedure), top 100 models (top 100, selected through further clustering) and top 10 final models (top 10). Fourth column shows the lowest RMSD values obtained in three prediction runs. For comparison purposes, the last column contains lowest RMSD values obtained in three prediction runs without contact information.

|  | contact info (run 1) | | | | | contact info (run 2) | | | | contact info (run 3) | | | | | | contact info (best result) | | | | | no contact info (best result) | | | |
| --- | --- | --- | --- | --- | --- | --- | --- | --- | --- | --- | --- | --- | --- | --- | --- | --- | --- | --- | --- | --- | --- | --- | --- | --- |
|  | **10k** | **1k** | **100** | **10** | **10k** | | **1k** | **100** | **10** | | **10k** | **1k** | **100** | **10** | **10k** | | **1k** | **100** | **10** | **10k** | | **1k** | **100** | **10** |
| 1ALV | 1,67 | 1,67 | 1,67 | 2,99 | 1,99 | | 2,28 | 2,43 | 2,85 | | 1,60 | 1,82 | 1,82 | 2,28 | **1,60** | | **1,67** | **1,67** | **2,28** | 1,68 | | 2,12 | 3,20 | 2,47 |
| 1B9K | 2,83 | 2,83 | 2,83 | 3,50 | 1,70 | | 2,90 | 3,10 | 3,63 | | 2,57 | 2,96 | 3,61 | 3,49 | **1,70** | | **2,83** | **2,83** | **3,49** | 3,38 | | 6,18 | 9,74 | 9,95 |
| 1CZZ | 1,78 | 1,87 | 2,21 | 2,72 | 1,76 | | 1,89 | 2,56 | 3,54 | | 1,77 | 1,81 | 2,12 | 3,11 | **1,76** | | **1,81** | **2,12** | **2,72** | 2,03 | | 2,33 | 2,58 | 3,21 |
| 1D1Z | 3,94 | 4,16 | 4,96 | 4,70 | 4,21 | | 4,83 | 5,95 | 6,52 | | 5,14 | 5,21 | 5,79 | 7,77 | **3,94** | | **4,16** | **4,96** | **4,70** | 5,11 | | 5,26 | 6,85 | 7,48 |
| 1EG3 | 4,65 | 6,35 | 6,88 | 13,43 | 3,77 | | 7,99 | 13,42 | 12,47 | | 4,37 | 5,29 | 5,33 | 6,80 | **3,77** | | **5,29** | **5,33** | **6,80** | 6,17 | | 8,44 | 12,43 | 15,02 |
| 1GO5 | 3,22 | 3,22 | 4,11 | 4,23 | 2,84 | | 2,84 | 4,42 | 5,20 | | 2,97 | 3,48 | 4,62 | 6,84 | **2,84** | | **2,84** | **4,11** | **4,23** | 2,89 | | 3,40 | 3,65 | 4,66 |
| 1GY7 | 2,33 | 2,78 | 3,13 | 3,21 | 2,62 | | 3,46 | 8,54 | 9,91 | | 2,27 | 2,27 | 2,80 | 4,71 | **2,27** | | **2,27** | **2,80** | **3,21** | 3,73 | | 4,59 | 12,19 | 13,71 |
| 1H1R | 2,95 | 2,95 | 5,22 | 5,20 | 3,07 | | 4,18 | 5,12 | 7,18 | | 2,75 | 3,74 | 3,74 | 5,26 | **2,75** | | **2,95** | **3,74** | **5,20** | 3,86 | | 4,92 | 7,09 | 6,55 |
| 1I2H | 2,64 | 3,27 | 5,06 | 6,09 | 2,43 | | 3,36 | 4,54 | 5,75 | | 3,19 | 3,37 | 4,90 | 4,76 | **2,43** | | **3,27** | **4,54** | **4,76** | 2,71 | | 3,33 | 3,95 | 4,31 |
| 1I7G | 1,14 | 1,14 | 1,75 | 2,33 | 1,27 | | 1,59 | 1,83 | 1,96 | | 1,60 | 1,60 | 1,70 | 2,68 | **1,14** | | **1,14** | **1,70** | **1,96** | 1,68 | | 1,83 | 1,83 | 1,87 |
| 1IE9 | 1,16 | 1,38 | 1,41 | 1,23 | 1,09 | | 1,63 | 1,63 | 2,17 | | 1,10 | 1,40 | 1,98 | 2,65 | **1,09** | | **1,38** | **1,41** | **1,23** | 4,85 | | 7,75 | 10,22 | 12,85 |
| 1JBE | 3,54 | 3,54 | 5,05 | 5,90 | 4,42 | | 5,16 | 7,70 | 6,67 | | 4,08 | 5,13 | 5,53 | 6,17 | **3,54** | | **3,54** | **5,05** | **5,90** | 4,37 | | 4,58 | 5,75 | 6,08 |
| 1JWF | 1,90 | 1,90 | 3,09 | 3,66 | 2,25 | | 2,41 | 4,29 | 5,18 | | 2,02 | 2,65 | 2,80 | 2,83 | **1,90** | | **1,90** | **2,80** | **2,83** | 2,17 | | 2,46 | 4,24 | 4,70 |
| 1JWT | 6,71 | 9,77 | 12,98 | 11,42 | 7,93 | | 8,25 | 9,76 | 6,73 | | 7,46 | 8,50 | 9,61 | 7,41 | **6,71** | | **8,25** | **9,61** | **6,73** | 8,68 | | 11,80 | 14,42 | 15,36 |
| 1LF7 | 2,85 | 3,90 | 3,90 | 4,87 | 3,15 | | 5,28 | 6,23 | 5,86 | | 3,97 | 3,97 | 5,91 | 6,09 | **2,85** | | **3,90** | **3,90** | **4,87** | 4,10 | | 4,97 | 4,98 | 4,83 |
| 1LVB | 2,87 | 3,77 | 5,37 | 4,16 | 2,05 | | 2,05 | 2,45 | 3,95 | | 1,17 | 1,90 | 2,43 | 2,93 | **1,17** | | **1,90** | **2,43** | **2,93** | 5,73 | | 7,12 | 13,21 | 17,56 |
| 1M7D | 2,22 | 2,59 | 2,59 | 3,19 | 1,79 | | 1,79 | 2,30 | 3,57 | | 1,74 | 2,13 | 2,23 | 2,97 | **1,74** | | **1,79** | **2,23** | **2,97** | 3,94 | | 4,67 | 5,50 | 5,89 |
| 1N7E | 2,84 | 3,30 | 3,54 | 5,78 | 1,52 | | 1,81 | 3,09 | 4,91 | | 2,54 | 3,22 | 3,49 | 5,61 | **1,52** | | **1,81** | **3,09** | **4,91** | 3,79 | | 4,79 | 8,03 | 11,26 |
| 1N83 | 0,91 | 1,21 | 1,52 | 2,59 | 1,06 | | 1,06 | 1,84 | 1,87 | | 0,94 | 2,03 | 2,46 | 2,50 | **0,91** | | **1,06** | **1,52** | **1,87** | 1,12 | | 1,13 | 1,13 | 2,39 |
| 1OEW | 4,61 | 6,46 | 6,56 | 10,81 | 2,72 | | 3,17 | 3,41 | 3,37 | | 3,49 | 3,67 | 3,79 | 3,74 | **2,72** | | **3,17** | **3,41** | **3,37** | 5,72 | | 6,58 | 7,84 | 9,00 |
| 1OOT | 3,12 | 3,12 | 3,66 | 6,03 | 3,50 | | 3,50 | 5,43 | 6,41 | | 3,48 | 4,43 | 4,71 | 5,90 | **3,12** | | **3,12** | **3,66** | **5,90** | 3,43 | | 4,62 | 5,73 | 6,90 |
| 1OU9 | 2,82 | 2,82 | 3,05 | 4,41 | 3,10 | | 3,59 | 4,73 | 5,05 | | 2,52 | 3,13 | 5,20 | 6,37 | **2,52** | | **2,82** | **3,05** | **4,41** | 3,25 | | 3,64 | 4,81 | 5,18 |
| 1PYW | 3,51 | 3,59 | 3,82 | 4,22 | 3,46 | | 3,46 | 4,02 | 4,03 | | 3,18 | 3,18 | 4,23 | 4,41 | **3,18** | | **3,18** | **3,82** | **4,03** | 3,62 | | 3,65 | 3,79 | 4,68 |
| 1QBH | 3,26 | 4,37 | 5,84 | 6,18 | 4,07 | | 4,07 | 5,13 | 5,43 | | 3,82 | 4,38 | 4,67 | 5,34 | **3,26** | | **4,07** | **4,67** | **5,34** | 4,72 | | 6,00 | 8,51 | 10,44 |
| 1R6J | 0,83 | 1,21 | 1,47 | 1,47 | 1,22 | | 1,24 | 1,39 | 2,52 | | 0,84 | 1,19 | 1,25 | 1,37 | **0,83** | | **1,19** | **1,25** | **1,37** | 2,52 | | 2,52 | 4,94 | 8,31 |
| 1RWZ | 4,19 | 4,19 | 5,81 | 5,44 | 2,01 | | 4,60 | 5,32 | 6,72 | | 3,52 | 4,13 | 5,26 | 7,22 | **2,01** | | **4,13** | **5,26** | **5,44** | 4,71 | | 4,71 | 6,67 | 7,61 |
| 1TQ3 | 0,87 | 0,87 | 1,23 | 1,44 | 1,03 | | 1,18 | 1,18 | 1,18 | | 0,74 | 0,91 | 1,11 | 1,76 | **0,74** | | **0,87** | **1,11** | **1,18** | 1,40 | | 1,40 | 3,57 | 6,44 |
| 1UM5 | 3,18 | 3,65 | 4,75 | 6,18 | 3,65 | | 4,20 | 5,15 | 4,99 | | 2,84 | 3,80 | 4,18 | 5,21 | **2,84** | | **3,65** | **4,18** | **4,99** | 3,05 | | 3,05 | 3,34 | 3,93 |
| 1UTN | 4,84 | 5,33 | 6,12 | 7,45 | 3,95 | | 4,16 | 6,67 | 5,88 | | 5,05 | 5,47 | 6,20 | 6,22 | **3,95** | | **4,16** | **6,12** | **5,88** | 6,95 | | 7,66 | 8,69 | 10,01 |
| 1V49 | 1,71 | 1,75 | 2,74 | 3,67 | 1,83 | | 1,99 | 2,60 | 3,54 | | 1,88 | 1,88 | 2,68 | 2,99 | **1,71** | | **1,75** | **2,60** | **2,99** | 2,19 | | 2,56 | 2,73 | 3,45 |
| 1X2J | 3,31 | 4,35 | 4,98 | 5,33 | 2,95 | | 4,26 | 5,05 | 4,23 | | 3,15 | 3,76 | 4,72 | 5,97 | **2,95** | | **3,76** | **4,72** | **4,23** | 3,44 | | 3,68 | 5,17 | 5,89 |
| 1Y0M | 2,97 | 3,08 | 3,64 | 4,36 | 3,18 | | 3,61 | 4,10 | 4,25 | | 2,86 | 3,53 | 4,43 | 5,00 | **2,86** | | **3,08** | **3,64** | **4,25** | 3,53 | | 4,83 | 5,45 | 6,21 |
| 1YEJ | 3,45 | 4,17 | 5,22 | 5,59 | 3,95 | | 3,95 | 5,49 | 6,18 | | 3,32 | 4,44 | 5,13 | 5,56 | **3,32** | | **3,95** | **5,13** | **5,56** | 4,05 | | 5,20 | 8,05 | 7,67 |
| 1Z1M | 3,07 | 3,50 | 3,75 | 5,10 | 3,07 | | 3,13 | 3,15 | 3,86 | | 3,17 | 3,47 | 3,86 | 4,48 | **3,07** | | **3,13** | **3,15** | **3,86** | 2,76 | | 3,46 | 3,72 | 3,62 |
| 1Z9L | 3,45 | 3,45 | 6,86 | 7,20 | 2,92 | | 2,92 | 3,60 | 4,00 | | 3,54 | 5,09 | 5,55 | 8,98 | **2,92** | | **2,92** | **3,60** | **4,00** | 3,80 | | 4,84 | 4,91 | 5,08 |
| 2AA2 | 1,11 | 2,25 | 2,92 | 3,18 | 1,97 | | 2,35 | 3,50 | 4,17 | | 1,73 | 3,34 | 4,40 | 5,59 | **1,11** | | **2,25** | **2,92** | **3,18** | 1,73 | | 2,00 | 2,54 | 2,95 |
| 2ABX | 5,88 | 6,59 | 8,74 | 9,21 | 5,80 | | 6,46 | 7,99 | 8,90 | | 6,33 | 8,39 | 11,90 | 13,33 | **5,80** | | **6,46** | **7,99** | **8,90** | 6,14 | | 7,22 | 7,96 | 8,76 |
| 2ALF | 1,65 | 1,65 | 1,65 | 3,05 | 1,82 | | 1,82 | 2,20 | 2,14 | | 1,58 | 2,09 | 2,59 | 2,61 | **1,58** | | **1,65** | **1,65** | **2,14** | 1,74 | | 1,81 | 2,70 | 2,55 |
| 2AM9 | 1,23 | 1,32 | 1,61 | 2,08 | 1,21 | | 1,23 | 1,23 | 2,00 | | 1,25 | 1,56 | 1,56 | 2,20 | **1,21** | | **1,23** | **1,23** | **2,00** | 1,21 | | 1,58 | 1,69 | 2,14 |
| 2B9F | 2,13 | 2,40 | 2,58 | 2,97 | 2,39 | | 2,39 | 2,39 | 2,47 | | 2,19 | 2,52 | 3,35 | 3,88 | **2,13** | | **2,39** | **2,39** | **2,47** | 2,33 | | 2,66 | 3,52 | 3,86 |
| 2BZ6 | 5,87 | 8,48 | 10,85 | 12,59 | 4,54 | | 5,90 | 6,58 | 6,70 | | 5,79 | 6,35 | 6,99 | 8,25 | **4,54** | | **5,90** | **6,58** | **6,70** | 6,00 | | 9,88 | 10,72 | 10,51 |
| 2DS7 | 1,66 | 3,40 | 4,45 | 4,38 | 1,49 | | 1,49 | 1,49 | 2,33 | | 2,80 | 2,80 | 5,48 | 7,08 | **1,49** | | **1,49** | **1,49** | **2,33** | 3,63 | | 5,64 | 10,38 | 11,24 |
| 2DVJ | 2,96 | 3,57 | 4,15 | 7,18 | 1,96 | | 1,96 | 3,89 | 4,24 | | 2,50 | 3,25 | 3,39 | 4,60 | **1,96** | | **1,96** | **3,39** | **4,24** | 2,82 | | 3,29 | 5,09 | 6,15 |
| 2E45 | 3,21 | 4,29 | 6,24 | 6,37 | 3,14 | | 3,71 | 5,15 | 7,65 | | 3,45 | 4,56 | 5,58 | 7,23 | **3,14** | | **3,71** | **5,15** | **6,37** | 3,38 | | 5,35 | 7,04 | 10,34 |
| 2F1W | 2,92 | 3,50 | 4,43 | 5,03 | 2,39 | | 3,48 | 4,19 | 6,09 | | 2,73 | 3,51 | 4,62 | 6,17 | **2,39** | | **3,48** | **4,19** | **5,03** | 2,82 | | 3,35 | 3,35 | 6,91 |
| 2FGQ | 5,17 | 5,17 | 8,83 | 8,91 | 5,11 | | 7,66 | 9,35 | 14,69 | | 5,90 | 5,90 | 7,31 | 11,92 | **5,11** | | **5,17** | **7,31** | **8,91** | 7,73 | | 13,38 | 14,87 | 14,82 |
| 2G6F | 3,08 | 3,86 | 5,01 | 5,70 | 3,30 | | 4,23 | 4,87 | 5,34 | | 3,16 | 3,38 | 4,84 | 5,54 | **3,08** | | **3,38** | **4,84** | **5,34** | 3,25 | | 3,44 | 5,08 | 4,94 |
| 2H14 | 1,82 | 1,82 | 2,04 | 3,40 | 1,90 | | 2,66 | 2,75 | 3,25 | | 1,75 | 2,05 | 2,75 | 3,53 | **1,75** | | **1,82** | **2,04** | **3,25** | 2,28 | | 2,28 | 3,09 | 4,26 |
| 2H3L | 2,61 | 2,83 | 3,25 | 3,36 | 2,65 | | 3,68 | 3,85 | 4,76 | | 3,06 | 3,21 | 3,21 | 6,68 | **2,61** | | **2,83** | **3,21** | **3,36** | 3,54 | | 4,66 | 4,71 | 9,11 |
| 2HPJ | 2,00 | 2,04 | 2,20 | 5,51 | 1,46 | | 1,46 | 2,60 | 2,77 | | 1,52 | 2,24 | 2,97 | 4,25 | **1,46** | | **1,46** | **2,20** | **2,77** | 1,64 | | 2,17 | 3,37 | 4,58 |
| 2HWQ | 0,88 | 0,98 | 1,09 | 1,40 | 0,83 | | 0,93 | 1,07 | 1,46 | | 1,06 | 1,21 | 1,26 | 1,19 | **0,83** | | **0,93** | **1,07** | **1,19** | 1,16 | | 1,17 | 1,36 | 1,49 |
| 2I3I | 2,03 | 2,03 | 2,30 | 2,27 | 2,38 | | 3,03 | 3,09 | 5,01 | | 2,34 | 2,77 | 2,77 | 4,33 | **2,03** | | **2,03** | **2,30** | **2,27** | 3,36 | | 4,03 | 6,82 | 9,40 |
| 2IOG | 7,10 | 7,27 | 7,70 | 8,01 | 7,31 | | 9,12 | 10,05 | 11,03 | | 8,66 | 11,60 | 17,16 | 13,21 | **7,10** | | **7,27** | **7,70** | **8,01** | 8,77 | | 10,35 | 13,32 | 20,05 |
| 2J2I | 2,71 | 3,25 | 4,44 | 5,64 | 2,56 | | 5,70 | 5,70 | 9,15 | | 2,68 | 3,38 | 4,03 | 4,97 | **2,56** | | **3,25** | **4,03** | **4,97** | 3,06 | | 4,03 | 8,73 | 11,10 |
| 2J6K | 2,94 | 3,13 | 3,94 | 4,48 | 2,89 | | 2,89 | 3,85 | 3,78 | | 2,59 | 3,27 | 4,53 | 4,82 | **2,59** | | **2,89** | **3,85** | **3,78** | 2,77 | | 3,06 | 3,34 | 4,03 |
| 2O9S | 2,91 | 3,43 | 3,43 | 4,17 | 3,16 | | 3,65 | 4,13 | 5,17 | | 2,81 | 3,84 | 4,98 | 5,47 | **2,81** | | **3,43** | **3,43** | **4,17** | 3,23 | | 3,86 | 5,01 | 6,36 |
| 2QBH | 3,39 | 3,95 | 4,30 | 4,95 | 4,27 | | 6,54 | 7,55 | 12,26 | | 3,41 | 3,90 | 4,02 | 5,03 | **3,39** | | **3,90** | **4,02** | **4,95** | 5,03 | | 5,03 | 7,98 | 10,33 |
| 2QHN | 1,43 | 1,43 | 1,72 | 2,26 | 1,83 | | 2,32 | 3,07 | 2,42 | | 1,91 | 2,34 | 2,91 | 3,78 | **1,43** | | **1,43** | **1,72** | **2,26** | 3,74 | | 3,87 | 7,10 | 5,22 |
| 2RTM | 4,42 | 5,61 | 6,45 | 7,02 | 4,62 | | 4,86 | 5,59 | 5,61 | | 4,19 | 6,56 | 9,42 | 10,61 | **4,19** | | **4,86** | **5,59** | **5,61** | 4,44 | | 5,13 | 5,96 | 6,49 |
| 2YQL | 2,55 | 4,93 | 5,09 | 7,08 | 1,71 | | 1,71 | 1,71 | 4,00 | | 1,88 | 2,44 | 3,05 | 3,77 | **1,71** | | **1,71** | **1,71** | **3,77** | 3,29 | | 3,29 | 6,49 | 7,59 |
| 3D1G | 1,64 | 1,82 | 3,60 | 3,43 | 1,97 | | 1,97 | 3,43 | 3,92 | | 1,47 | 2,44 | 3,76 | 3,29 | **1,47** | | **1,82** | **3,43** | **3,29** | 2,73 | | 2,73 | 7,04 | 6,88 |
| 3EKK | 4,72 | 6,78 | 6,78 | 8,72 | 3,39 | | 3,49 | 5,93 | 4,75 | | 4,91 | 4,91 | 6,07 | 6,66 | **3,39** | | **3,49** | **5,93** | **4,75** | 4,41 | | 6,57 | 7,11 | 8,56 |
| 3HAU | 5,16 | 5,59 | 6,36 | 6,30 | 2,53 | | 2,60 | 2,64 | 2,85 | | 3,12 | 3,26 | 3,40 | 3,54 | **2,53** | | **2,60** | **2,64** | **2,85** | 6,24 | | 6,24 | 6,80 | 7,00 |
| 3NSQ | 2,35 | 3,47 | 3,74 | 6,25 | 3,24 | | 3,44 | 5,78 | 8,15 | | 2,48 | 2,54 | 3,55 | 3,15 | **2,35** | | **2,54** | **3,55** | **3,15** | 2,40 | | 2,64 | 3,76 | 5,76 |
| 3POM | 1,75 | 2,39 | 3,20 | 4,12 | 2,54 | | 3,19 | 4,45 | 4,48 | | 2,09 | 2,09 | 3,48 | 3,03 | **1,75** | | **2,09** | **3,20** | **3,03** | 2,17 | | 2,17 | 3,59 | 6,42 |
| 3RDH | 3,61 | 3,78 | 4,29 | 4,80 | 3,42 | | 3,42 | 4,23 | 4,07 | | 3,02 | 3,75 | 4,69 | 5,13 | **3,02** | | **3,42** | **4,23** | **4,07** | 3,58 | | 3,91 | 4,24 | 4,96 |
| 3SIQ | 4,23 | 5,56 | 6,96 | 9,34 | 2,85 | | 2,85 | 4,41 | 5,92 | | 3,87 | 3,87 | 5,70 | 8,77 | **2,85** | | **2,85** | **4,41** | **5,92** | 5,08 | | 5,08 | 8,65 | 10,19 |
| 3TX7 | 2,00 | 2,78 | 2,78 | 3,58 | 2,67 | | 3,14 | 4,28 | 5,11 | | 3,35 | 4,53 | 9,66 | 10,06 | **2,00** | | **2,78** | **2,78** | **3,58** | 3,22 | | 3,69 | 5,28 | 8,25 |
| **MEAN** | **2,95** | **3,51** | **4,32** | **5,19** | **2,83** | | **3,43** | **4,42** | **5,12** | | **2,97** | **3,57** | **4,49** | **5,31** | **2,57** | | **2,96** | **3,64** | **4,10** | **3,68** | | **4,49** | **6,14** | **7,32** |
